# Efficacy and safety of sarilumab in combination with csDMARDs or as monotherapy in subpopulations of patients with moderately to severely active rheumatoid arthritis in three phase III randomized, controlled studies

**DOI:** 10.1186/s13075-020-02194-z

**Published:** 2020-06-10

**Authors:** Mark C. Genovese, Roy Fleischmann, Alan Kivitz, Eun-Bong Lee, Hubert van Hoogstraten, Toshio Kimura, Gregory St John, Erin K. Mangan, Gerd R. Burmester

**Affiliations:** 1 Division of Immunology and Rheumatology, Stanford University School of Medicine, Palo Alto, CA USA; 2 University of Texas Southwestern and Metroplex Clinical Research Center, Dallas, TX USA; 3 Altoona Center for Clinical Research, Duncansville, PA USA; 4 Seoul National University College of Medicine, Seoul, South Korea; 5 Sanofi, Bridgewater, NJ USA; 6 Regeneron Pharmaceuticals, Inc., Tarrytown, NY USA; 7 Charité-University Medicine Berlin, Free University and Humboldt University Berlin, Berlin, Germany

**Keywords:** Rheumatoid arthritis, Sarilumab, Interleukin-6, Adalimumab, csDMARDs, Methotrexate, Subpopulations

## Abstract

**Background:**

The interleukin-6 receptor inhibitor sarilumab demonstrated efficacy in combination with conventional synthetic disease-modifying antirheumatic drugs (csDMARDs) or as monotherapy in patients with moderately to severely active rheumatoid arthritis (RA) with an inadequate response (IR) or intolerant (INT) to methotrexate (MTX) or tumour necrosis factor (TNF)-α inhibitors. This analysis investigated the efficacy and safety of sarilumab in patient subgroups.

**Methods:**

Data were included from phase III studies: two placebo-controlled studies of subcutaneous sarilumab 150/200 mg every 2 weeks (q2w) either + MTX in MTX-IR patients (52 weeks) or + csDMARDs in TNF-IR/INT patients (24 weeks), and a monotherapy study of sarilumab 200 mg q2w vs. adalimumab 40 mg q2w in MTX-IR/INT patients (24 weeks). Prespecified and post hoc subgroups included patient demographics, disease characteristics, and prior treatments. Prespecified and post hoc endpoints included clinical, radiographic, and physical function measures, and *p* values are considered nominal. Safety was assessed during double-blind treatment.

**Results:**

The superiority of sarilumab (either as monotherapy vs. adalimumab or in combination with csDMARDs vs. placebo + csDMARDs) across clinical endpoints was generally consistent across subgroups defined by patient demographics, disease characteristics, and prior treatments, demonstrating the benefit of sarilumab treatment for a wide range of patient types. Interaction *p* values of < 0.05 were consistently observed across studies only for baseline anti-cyclic citrullinated peptide antibody (ACPA) status for American College of Rheumatology 20% response, but not American College of Rheumatology 50% or 70% response. Adverse events and worsening laboratory parameters occurred more frequently in sarilumab-treated vs. placebo-treated patients and were more frequent in the small number of patients ≥ 65 years (*n* = 289) vs. patients < 65 years (*n* = 1819). Serious infections occurred in six patients aged ≥ 65 years receiving sarilumab, although the incidence of serious infections was generally higher in patients aged ≥ 65 years regardless of treatment.

**Conclusions:**

Apart from ACPA status, there were no consistent signals indicating differential effects of sarilumab in any of the subpopulations assessed. Sarilumab demonstrated consistent efficacy and safety across a wide range of patients with RA.

**Trial registration:**

ClinicalTrials.gov NCT01061736, registered on February 03, 2010; ClinicalTrials.gov NCT01709578, registered on October 18, 2012; ClinicalTrials.gov NCT02332590, registered on January 07, 2015

## Background

Rheumatoid arthritis (RA) affects nearly 20 million people worldwide [1] and is associated with substantial morbidity and disability [2]. The consequences of RA disease progression on patient health may be influenced by multiple genetic and environmental factors [3], and specific subpopulations of patients with RA can be characterized on the basis of differences in demographic variables, clinical features, and biomarkers, some of which have been shown to be associated with disease outcomes and therapeutic responses [4–9].

Characterization of subpopulations of patients most likely to respond favourably or unfavourably to conventional synthetic disease-modifying antirheumatic drugs (csDMARDs) and/or biologic DMARDs (bDMARDs) could help guide appropriate use of the many therapies now available and improve patient outcomes. Obesity and current smoking are independent predictors of failure to achieve adequate disease control after the first or second csDMARD [10]. Studies have suggested that the presence of anti-cyclic citrullinated peptide antibodies (ACPA) may be associated with superior responses to rituximab, adalimumab, and abatacept [11, 12], but also increased mortality [13], and may mark an indication for more intense RA treatment with csDMARDs and glucocorticoids [14]. Furthermore, the European League Against Rheumatism (EULAR) RA management recommendations list the presence of rheumatoid factor (RF) and/or ACPA as poor prognostic factors and recommend that, if present, patients are treated with a bDMARD after failure to achieve the target with initial methotrexate (MTX) treatment [15]. However previous approaches to define useful baseline characteristics that predict treatment response have been largely unsuccessful due to inconsistent findings, low predictive value, and lack of validation [16].

Sarilumab is a human monoclonal antibody that binds membrane-bound and soluble interleukin-6 (IL-6) receptor-α to inhibit IL-6 signalling. Sarilumab is approved as a monotherapy or in combination with csDMARDs for the treatment of adults with moderately to severely active RA with an inadequate response (IR) or intolerant (INT) to one or more csDMARDs. In phase III clinical trials, sarilumab has demonstrated efficacy in patients with IR or who are INT to csDMARDs, including MTX, and to tumour necrosis factor (TNF)-α inhibitors, and it has demonstrated superiority as monotherapy vs. adalimumab monotherapy for improving the signs and symptoms of RA and physical function in patients with IR or who are INT to MTX [17–19].

Here, we explore the efficacy, safety, and consistency of treatment effects of sarilumab vs. placebo or adalimumab across a range of predefined and post hoc patient subpopulations in three phase III trials of sarilumab in patients with RA.

## Methods

### Study designs

MOBILITY (NCT01061736; subsequently defined as the MTX-IR combination study), TARGET (NCT01709578; subsequently defined as the TNF-IR/INT combination study), and MONARCH (NCT02332590; subsequently defined as the monotherapy study) were phase III multicentre, randomized, controlled studies (RCTs). Study protocols were approved by appropriate ethics committees/institutional review boards, and the trials were conducted in accordance with the International Conference on Harmonisation Guidelines for Good Clinical Practice and the Declaration of Helsinki. All patients provided written informed consent before the initiation of study procedures.

The study designs and patient eligibility criteria have been described in full elsewhere [17–19]. In brief, the MTX-IR combination study investigated subcutaneous (SC) sarilumab 150 or 200 mg every 2 weeks (q2w) or placebo (1:1:1 randomization) in combination with weekly MTX for 52 weeks in adults with moderately to severely active RA with MTX-IR [19]. In the TNF-IR/INT combination study, adults with TNF-IR or who were INT, with moderately to severely active RA, were randomized (1:1:1) to receive SC sarilumab 150 or 200 mg q2w or placebo q2w in combination with background csDMARDs for 24 weeks [18]. In the monotherapy study, bDMARD-naive adults with MTX-IR or who were INT, with moderately to severely active RA, were randomized (1:1) to receive SC sarilumab 200 mg q2w or SC adalimumab 40 mg q2w for 24 weeks [17].

### Patient subpopulations

The baseline characteristics prespecified in the individual trial protocols for efficacy analyses stratified by patient subpopulations were age, sex, race (classified by the investigator), region, weight, body mass index (BMI), smoking history, duration of RA, baseline RF and ACPA status, and levels of C-reactive protein (CRP). The following were also prespecified for individual studies: number of prior csDMARDs (MTX-IR and TNF-IR/INT combination studies), prior bDMARD use (MTX-IR combination study), number of previous anti-TNFs, MTX vs. non-MTX use, background csDMARD use (TNF-IR/INT combination study), baseline erythrocyte sedimentation rate (ESR), and MTX IR vs. INT/inappropriate (monotherapy study). Further details of the subgroups, as well as exploratory analyses performed post hoc for all 3 studies, are described in Additional file 1.

### Endpoints

#### Primary endpoints

Efficacy was assessed in patient subpopulations for the coprimary/primary endpoints of each study: American College of Rheumatology 20% (ACR20) response at week 24, change from baseline in the Health Assessment Questionnaire-Disability Index (HAQ-DI) at week 16, and change from baseline in the modified total Sharp/van der Heijde score (mTSS) at week 52 for the MTX-IR combination study; ACR20 at week 24 and change from baseline in the HAQ-DI at week 12 for the TNF-IR/INT combination study; and change from baseline in the Disease Activity Score in 28 joints using ESR (DAS28-ESR) at week 24 for the monotherapy study.

#### Secondary endpoints

Secondary efficacy endpoints for subanalyses at week 24 included ACR20 (monotherapy study), ACR 50% response (ACR50), and ACR 70% response (ACR70); DAS28-ESR remission (< 2.6; monotherapy study only); mean change from baseline in DAS28 using CRP (DAS28-CRP); proportion of patients achieving DAS28-CRP < 2.6; mean change from baseline in HAQ-DI ≥ 0.22 (established threshold for minimal clinically important difference [20]) and ≥ 0.3 [17, 18, 20–22] units of improvement in HAQ-DI; mean change in Clinical Disease Activity Index (CDAI) and Simplified Disease Activity Index (SDAI) from baseline to week 24; CDAI remission (≤ 2.8); and SDAI remission (≤ 3.3).

Improvements from baseline ≥ 58% and ≥ 85% in CDAI and SDAI were defined post hoc as exploratory subanalyses, as they have been shown at week 12 to sensitively predict low disease activity (≥ 58%) and remission (≥ 85%) at 6 months [23].

Safety was reported as the occurrence of treatment-emergent adverse events (TEAEs), serious TEAEs (SAEs), serious infections, and specific abnormalities in laboratory parameters. Adverse events (AEs) were described at the Medical Dictionary for Regulatory Activities (version 16.0) preferred term level. Proportions of patients with grade 3 (absolute neutrophil count [ANC] ≥ 0.5–1 × 10^9^/L) and grade 4 (ANC < 0.5 × 10^9^/L) neutropenia were assessed.

### Statistical analyses

Subanalyses of efficacy and safety by patient subpopulation were conducted with the intention-to-treat and safety populations, respectively. In the sarilumab monotherapy study, the comparison of sarilumab 200 mg q2w with adalimumab included a minority of patients (*n* = 16), who increased adalimumab dose to 40 mg every week, in addition to those receiving 40 mg q2w (*n* = 169).

For categorical efficacy variables, patients were considered non-responders from the time they discontinued study medication or started rescue medication; missing values were set to non-response. Mantel-Haenszel estimates of the odds ratio (OR) with corresponding 95% confidence intervals (CIs), stratified by region and prior bDMARDs (MTX-IR combination study only) or prior anti-TNFs (TNF-IR/INT combination study only), were derived by testing each treatment group separately by subpopulation.

Treatment-by-subgroup interaction was tested using logistic regression with baseline and terms of treatment, study stratification variables, subpopulation, and treatment-by-subpopulation. The interaction *p* value from treatment-by-subgroup or from treatment-by-visit-by-subpopulation at the visit of interest was used to assess the treatment effect differences across subpopulations. *p* values for all analyses should be considered nominal. Analysis of safety by age was also conducted post hoc.

For continuous efficacy variables, assessments were set to missing from the time a patient discontinued study medication early or received rescue medication; missing values were not imputed. The least-squares mean (LSM) difference and corresponding 95% CIs were derived from a mixed-effects model for repeated measures, assuming an unstructured covariance structure with covariate baseline and terms of treatment, study stratification variables, subpopulation, treatment-by-subpopulation, visit, treatment-by-visit, and treatment-by-visit-by-subpopulation.

## Results

### Patients

Baseline demographics and patient characteristics for the three study populations have been reported previously [17–19] and were generally well balanced between the treatment groups in each individual study and are summarized in Table S1 (see Additional file 1).

### Efficacy

**Fig. 1:**
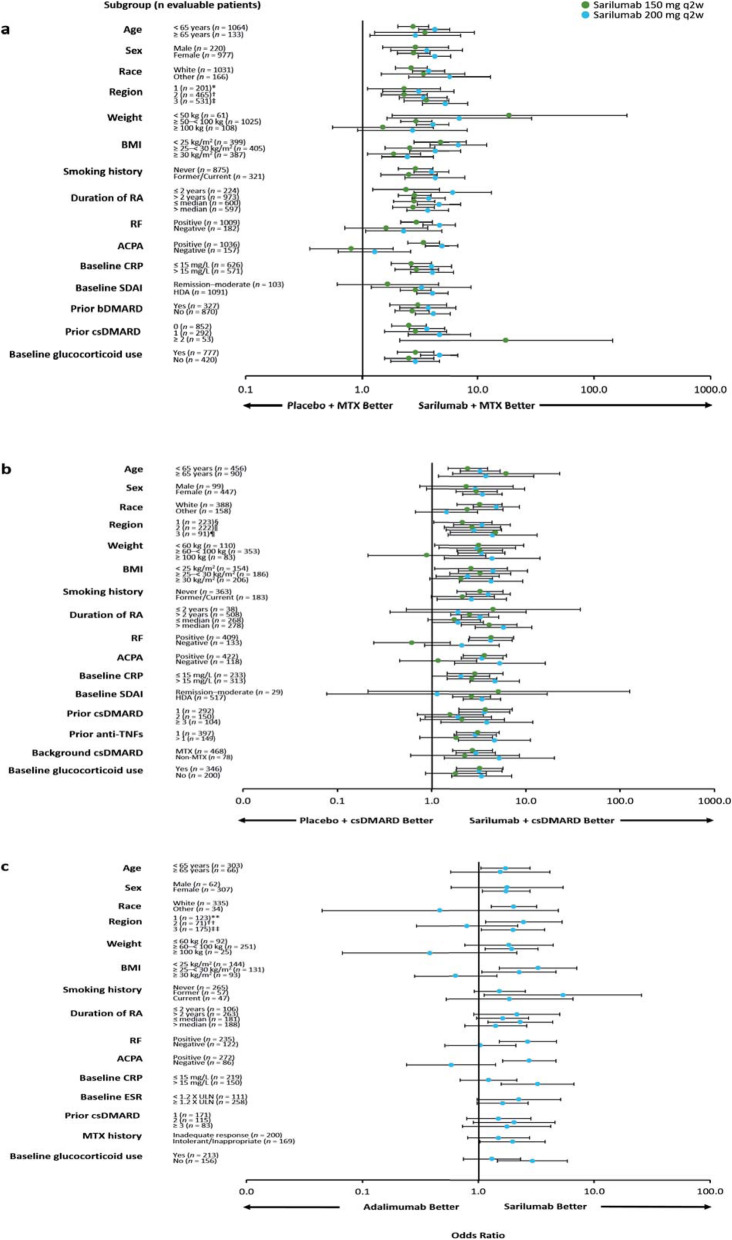
Odds ratio (95% CI) for ACR20 response by subpopulation at week 24. **a** Sarilumab 150/200 mg q2w + MTX vs. placebo + MTX in MTX-IR patients. **b** Sarilumab 150/200 mg q2w + csDMARDs vs. placebo + csDMARDs in TNF-IR/INT patients. **c** Sarilumab 200 mg q2w vs. adalimumab 40 mg q2w in MTX-IR/INT patients. Mantel-Haenszel estimate with terms of treatment: **a** treatment, prior biologic use, region, subpopulation, and treatment-by-subpopulation; **b** treatment, prior anti-TNF use, region, subpopulation, and treatment-by-subpopulation; and **c** treatment, region, subpopulation, and treatment-by-subpopulation. ACPA, anti-cyclic citrullinated peptide antibody; ACR20, American College of Rheumatology 20% response; bDMARD, biological and targeted disease-modifying antirheumatic drug; BMI, body mass index; CI, confidence interval; CRP, C-reactive protein; csDMARD, conventional synthetic disease-modifying antirheumatic drug; ESR, erythrocyte sedimentation rate; HDA, high disease activity; INT, intolerant; IR, inadequate response; MTX, methotrexate; *n*, number of evaluable patients regardless of the treatment group; q2w, every 2 weeks; RA, rheumatoid arthritis; RF, rheumatoid factor; SDAI, Simplified Disease Activity Index; TNF, tumour necrosis factor; ULN, upper limit of normal. *Austria, Australia, Belgium, Canada, Finland, Germany, Greece, Hungary, New Zealand, Norway, Portugal, Spain, and USA; ^†^Argentina, Brazil, Chile, Colombia, and Mexico; ^‡^Belarus, Estonia, India, Malaysia, Philippines, Poland, Romania, Russia, South Africa, South Korea, Taiwan, Thailand, and Ukraine; ^§^Australia, Canada, Czech Republic, Germany, Greece, Hungary, Israel, Italy, New Zealand, Portugal, Spain, and USA; ^‖^Argentina, Brazil, Chile, Colombia, Ecuador, Guatemala, Mexico, and Peru; ^¶^South Korea, Lithuania, Poland, Russia, Taiwan, Turkey, and Ukraine; **Czech Republic, Germany, Hungary, Israel, Spain, and USA; ^††^Chile and Peru; ^‡‡^South Korea, Poland, South Africa, Romania, Russia, and Ukraine

**Fig. 2:**
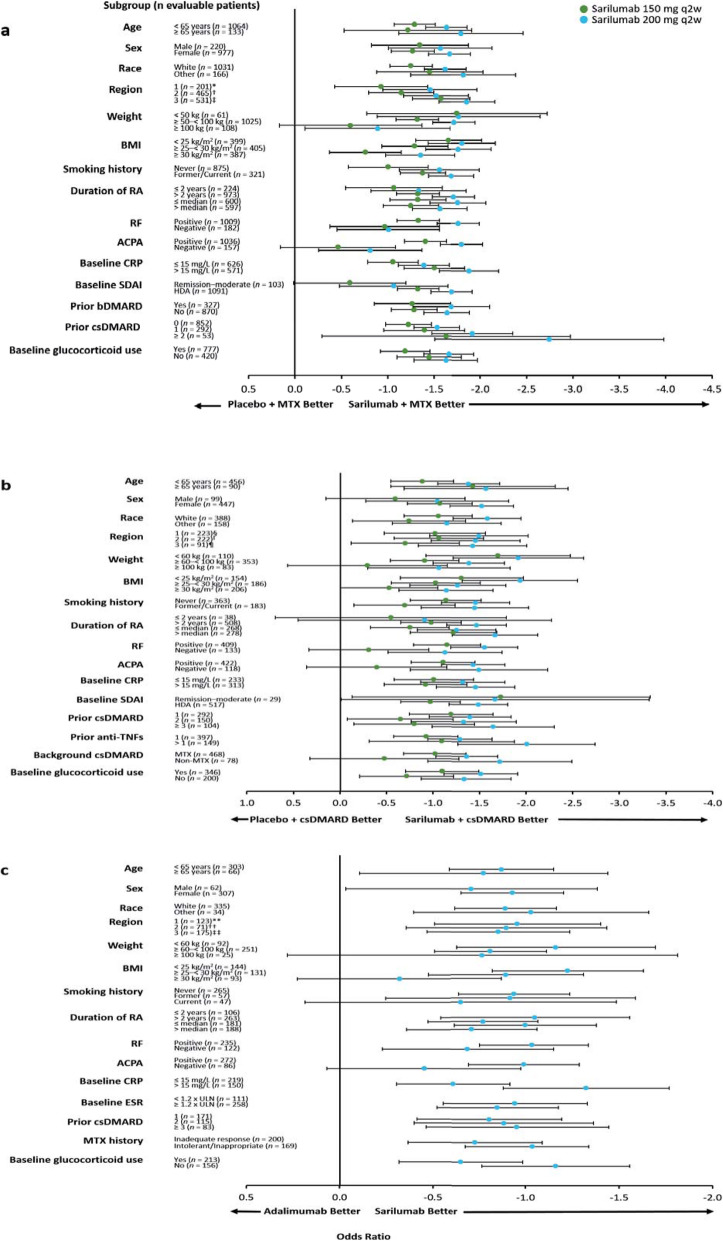
LSM (95% CI) treatment difference for change from baseline in DAS28-CRP at week 24. **a** Sarilumab 150/200 mg q2w + MTX vs. placebo + MTX in MTX-IR patients. **b** Sarilumab 150/200 mg q2w + csDMARDs vs. placebo + csDMARDs in TNF-IR/INT patients. **c** Sarilumab 200 q2w vs. adalimumab 40 mg q2w in MTX-IR/INT patients. Mixed-effect model for repeated measures with PROC MIXED assuming an unstructured covariance structure: **a** baseline, treatment, prior biologic use, region, visit, and treatment-by-visit interaction; **b** baseline, treatment, prior anti-TNF use, region, visit, and treatment-by-visit interaction; and **c** baseline, treatment, region, visit, and treatment-by-visit interaction. ACPA, anti-cyclic citrullinated peptide antibody; bDMARD, biological and targeted disease-modifying antirheumatic drug; BMI, body mass index; CI, confidence interval; CRP, C-reactive protein; csDMARD, conventional synthetic disease-modifying antirheumatic drug; DAS28-CRP, Disease Activity Score in 28 joints using CRP; ESR, erythrocyte sedimentation rate; HDA, high disease activity; INT, intolerant; IR, inadequate response; LSM, least squares mean; MTX, methotrexate; *n*, number of evaluable patients regardless of the treatment group; q2w, every 2 weeks; RA, rheumatoid arthritis; RF, rheumatoid factor; SDAI, Simplified Disease Activity Index; TNF, tumour necrosis factor; ULN, upper limit of normal. *Austria, Australia, Belgium, Canada, Finland, Germany, Greece, Hungary, New Zealand, Norway, Portugal, Spain, and USA; ^†^Argentina, Brazil, Chile, Colombia, and Mexico; ^‡^Belarus, Estonia, India, Malaysia, Philippines, Poland, Romania, Russia, South Africa, South Korea, Taiwan, Thailand, and Ukraine; ^§^Australia, Canada, Czech Republic, Germany, Greece, Hungary, Israel, Italy, New Zealand, Portugal, Spain, and USA; ^‖^Argentina, Brazil, Chile, Colombia, Ecuador, Guatemala, Mexico, and Peru; ^¶^South Korea, Lithuania, Poland, Russia, Taiwan, Turkey, and Ukraine; **Czech Republic, Germany, Hungary, Israel, Spain, and USA; ^††^Chile and Peru; ^‡‡^South Korea, Poland, South Africa, Romania, Russia, and Ukraine

**Fig. 3:**
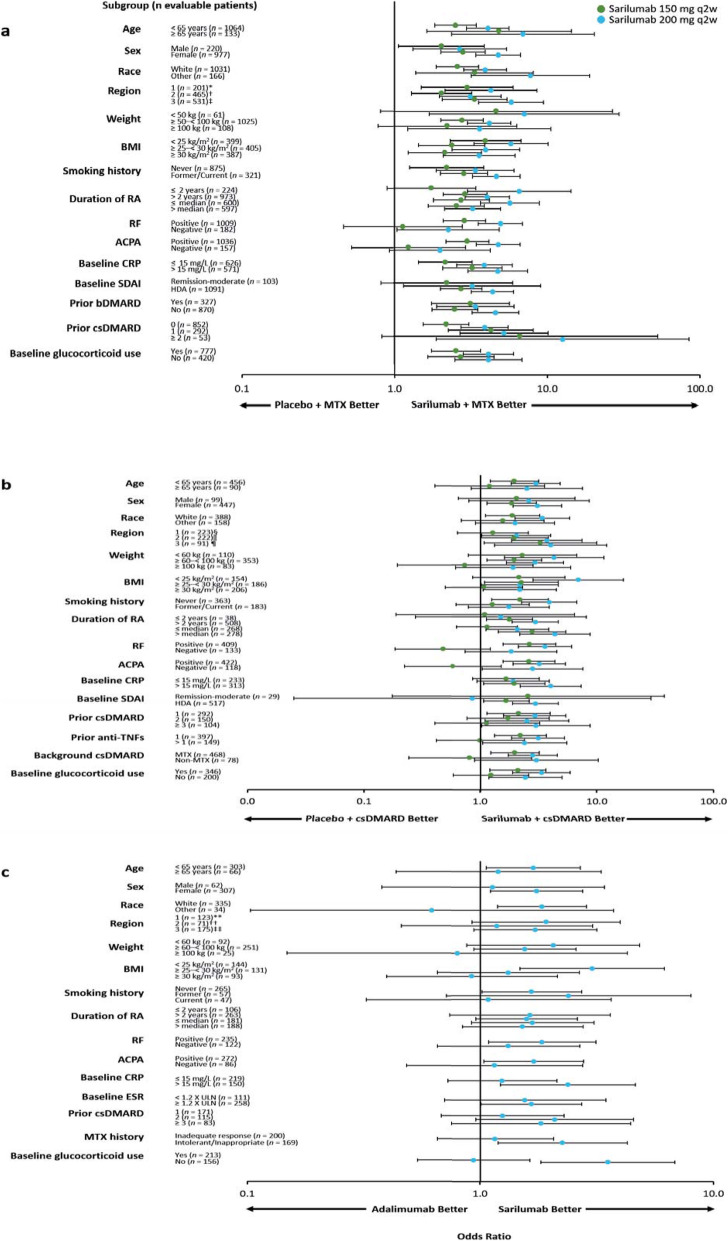
Odds ratio (95% CI) for improvement in CDAI ≥58% at week 24 by subpopulation. **a** Sarilumab 150/200 mg q2w + MTX vs. placebo + MTX in MTX-IR patients. **b** Sarilumab 150/200 mg q2w + csDMARDs vs. placebo + csDMARDs in TNF-IR/INT patients. **c** Sarilumab 200 mg q2w vs. adalimumab 40 mg q2w in MTX-IR/INT patients. Logistic regression model with terms of **a** treatment, prior biologic use, region, subpopulation, and treatment-by-subpopulation; **b** treatment, prior anti-TNF use, region, subpopulation, and treatment-by-subpopulation; and **c** treatment, region, subpopulation, and treatment-by-subpopulation. ACPA, anti-cyclic citrullinated peptide antibody; bDMARD, biological and targeted disease-modifying antirheumatic drug; BMI, body mass index; CI, confidence interval; CRP, C-reactive protein; csDMARD, conventional synthetic disease-modifying antirheumatic drug; CDAI, Clinical Disease Activity Index; ESR, erythrocyte sedimentation rate; HDA, high disease activity; INT, intolerant; IR, inadequate response; MTX, methotrexate; *n*, number of evaluable patients regardless of the treatment group; q2w, every 2 weeks; RA, rheumatoid arthritis; RF, rheumatoid factor; SDAI, Simplified Disease Activity Index; TNF, tumour necrosis factor; ULN, upper limit of normal. *Austria, Australia, Belgium, Canada, Finland, Germany, Greece, Hungary, New Zealand, Norway, Portugal, Spain, and USA; ^†^Argentina, Brazil, Chile, Colombia, and Mexico; ^‡^Belarus, Estonia, India, Malaysia, Philippines, Poland, Romania, Russia, South Africa, South Korea, Taiwan, Thailand, and Ukraine; ^§^Australia, Canada, Czech Republic, Germany, Greece, Hungary, Israel, Italy, New Zealand, Portugal, Spain, and USA; ^‖^Argentina, Brazil, Chile, Colombia, Ecuador, Guatemala, Mexico, and Peru; ^¶^South Korea, Lithuania, Poland, Russia, Taiwan, Turkey, and Ukraine; **Czech Republic, Germany, Hungary, Israel, Spain, and USA; ^††^Chile and Peru; ^‡‡^South Korea, Poland, South Africa, Romania, Russia, and Ukraine

**Fig. 4:**
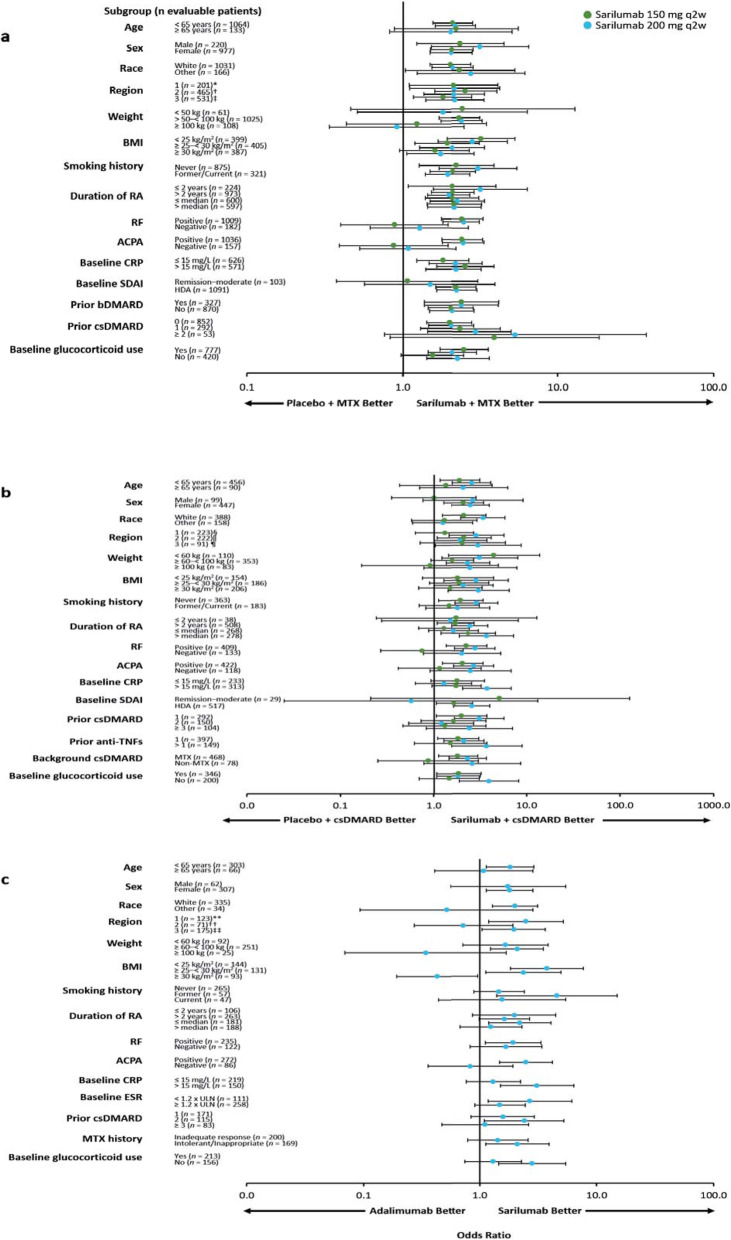
Odds ratio (95% CI) for HAQ-DI improvement ≥ 0.22 units at week 24 by subpopulation. **a** Sarilumab 150/200 mg q2w + MTX vs. placebo + MTX in MTX-IR patients. **b** Sarilumab 150/200 mg q2w + csDMARDs vs. placebo + csDMARDs in TNF-IR/INT patients. **c** Sarilumab 200 mg q2w vs. adalimumab 40 mg q2w in MTX-IR/INT patients. Logistic regression model with terms of **a** treatment, prior biologic use, region, subpopulation, and treatment-by-subpopulation; **b** treatment, prior anti-TNF use, region, subpopulation, and treatment-by-subpopulation; and **c** treatment, region, subpopulation, and treatment-by-subpopulation. ACPA, anti-cyclic citrullinated peptide antibody; bDMARD, biological and targeted disease-modifying antirheumatic drug; BMI, body mass index; CI, confidence interval; CRP, C-reactive protein; csDMARD, conventional synthetic disease-modifying antirheumatic drug; ESR, erythrocyte sedimentation rate; HAQ-DI, Health Assessment Questionnaire-Disability Index; HDA, high disease activity; INT, intolerant; IR, inadequate response; MTX, methotrexate; *n*, number of evaluable patients regardless of the treatment group; q2w, every 2 weeks; RA, rheumatoid arthritis; RF, rheumatoid factor; SDAI, Simplified Disease Activity Index; TNF, tumour necrosis factor; ULN, upper limit of normal. *Austria, Australia, Belgium, Canada, Finland, Germany, Greece, Hungary, New Zealand, Norway, Portugal, Spain, and USA; ^†^Argentina, Brazil, Chile, Colombia, and Mexico; ^‡^Belarus, Estonia, India, Malaysia, Philippines, Poland, Romania, Russia, South Africa, South Korea, Taiwan, Thailand, and Ukraine; ^§^Australia, Canada, Czech Republic, Germany, Greece, Hungary, Israel, Italy, New Zealand, Portugal, Spain, and USA; ^‖^Argentina, Brazil, Chile, Colombia, Ecuador, Guatemala, Mexico, and Peru; ^¶^South Korea, Lithuania, Poland, Russia, Taiwan, Turkey, and Ukraine; **Czech Republic, Germany, Hungary, Israel, Spain, and USA; ^††^Chile and Peru; ^‡‡^South Korea, Poland, South Africa, Romania, Russia, and Ukraine

The superiority of sarilumab 150/200 mg q2w + MTX/csDMARDs vs. placebo + MTX/csDMARDs and of sarilumab 200 mg monotherapy vs. adalimumab monotherapy in the overall study populations has been previously reported for the prespecified primary and secondary endpoints [17–19]. The efficacy of sarilumab (+ csDMARDs or as monotherapy) in patient subgroups is described in Figs. 1, 2, 3, and 4 and Figures S1–S6 (see Additional file 1). Treatment interaction *p* values are shown in Table 1.

**Table 1:** Treatment-by-subpopulation interactions across all three trials: endpoints at week 24 (unless otherwise stated)

	ACR20	ACR50	ACR70	Change in DAS28-CRP	Change in DAS28-ESR	Change in CDAI	CDAI ≤ 2.8	CDAI ≥ 58%	HAQ-DI ≥ 0.22	Change in HAQ-DI*	mTSS**
Age											
MTX-IR combination study	0.4673	0.6419	0.8516	0.7974	–	0.7638	0.9784	0.4889	0.9232	0.5847	0.5570
TNF-IR/INT combination study	0.4095	0.7902	0.2886	0.4695	–	0.6915	0.1337	0.5813	0.8173	0.4101	–
Monotherapy study	0.9861	0.6249	0.8440	0.9712	0.8819	0.5079	0.5501	0.6872	0.3983	0.7994	–
Sex											
MTX-IR combination study	0.7934	0.7803	0.0643	NC	–	0.3802	0.4315	0.3500	0.6165	0.4633	0.5890
TNF-IR/INT combination study	0.6818	0.2610	0.2086	0.4134	–	0.5132	0.0614	0.7993	0.2999	**0.0417**	–
Monotherapy study	0.7733	0.2587	0.0944	0.6394	0.1953	0.6325	0.6609	0.6818	0.8029	0.1981	–
Race											
MTX-IR combination study	0.4769	0.4827	0.3125	0.7798	–	0.5666	0.5479	0.3820	0.7486	0.2893	0.6911
TNF-IR/INT combination study	**0.0337**	**0.0154**	0.0529	0.3539	–	0.3740	**0.0035**	0.4085	0.1012	0.6925	–
Monotherapy study	0.2497	0.5923	0.9450	0.6376	0.6242	0.8933	0.9985	0.3730	0.1100	0.6447	–
Region											
MTX-IR combination study	0.5715	0.7835	0.7406	0.2041	–	0.1234	0.3924	0.4628	0.8182	0.3900	0.4490
TNF-IR/INT combination study	0.7319	0.5572	0.3057	0.9603	–	0.9703	0.4381	0.5830	0.5106	0.9414	–
Monotherapy study	0.1965	0.2636	0.5468	0.9506	0.6213	0.8280	0.9865	0.7148	0.1223	0.1202	–
Weight											
MTX-IR combination study	0.5261	0.6026	0.2398	NC	–	NC	0.7471	0.9225	0.4171	NC	0.3874
TNF-IR/INT combination study	0.2172	0.9392	0.0593	0.2256	–	0.7918	0.1423	0.5359	0.2759	0.2538	–
Monotherapy study	0.2376	0.4871	0.1161	0.3297	0.2533	0.1991	0.9981	0.5408	0.0939	0.2366	–
BMI											
MTX-IR combination study	0.0539	0.7636	0.4607	**0.0166**	–	0.1116	0.8669	0.5427	0.3684	0.6091	0.1167
TNF-IR/INT combination study	0.3219	0.5705	0.4019	0.1699	–	0.5089	0.4425	0.0593	0.8816	0.4860	–
Monotherapy study	**0.0048**	**0.0186**	**0.0117**	**0.0231**	**0.0466**	**0.0239**	0.0608	**0.0492**	**<0.0001**	**0.0023**	–
Smoking history											
MTX-IR combination study	0.8811	0.7700	0.5258	NC	–	0.9072	0.4806	0.5732	0.4258	0.1465	0.2046
TNF-IR/INT combination study	0.4588	0.9214	0.3633	0.2900	–	0.2582	0.3958	0.1818	0.6147	0.7203	–
Monotherapy study	0.2545	0.4051	0.3061	0.6974	0.3829	0.4994	0.6053	0.7733	0.1321	0.4093	–
Duration RA (</≥ 2 years)											
MTX-IR combination study	0.5211	0.4003	0.1108	0.3018	–	0.0611	0.3217	0.1550	0.3796	0.3297	NC
TNF-IR/INT combination study	0.7880	0.8392	0.0778	0.7489	–	0.8370	0.2069	0.9211	0.9795	0.8628	–
Monotherapy study	0.6300	0.8484	0.6729	0.2816	0.4205	0.5917	0.5604	0.9527	0.7008	0.0512	–
Duration RA (</≥ median)											
MTX-IR combination study	0.8364	0.5116	0.1697	0.7286	–	0.7994	0.1961	0.1578	0.9931	0.5678	0.3215
TNF-IR/INT combination study	0.0516	0.1992	0.1678	0.3154	–	0.2261	0.2482	0.0951	0.2263	0.1228	–
Monotherapy study	0.2995	0.8856	0.8174	0.2916	0.8227	0.6843	0.4439	0.8276	0.2054	0.3787	–
RF											
MTX-IR combination study	0.1083	0.3854	0.0564	0.0551	–	0.6429	0.1869	0.0766	0.0721	0.0427	0.8194
TNF-IR/INT combination study	**0.0012**	**0.0480**	0.3007	0.0887	–	0.0528	0.2175	**0.0070**	0.0996	**0.0202**	–
Monotherapy study	**0.0416**	0.6937	0.7953	0.2139	0.6410	0.1476	0.4715	0.4768	0.7497	0.7273	–
ACPA											
MTX-IR combination study	**0.0010**	0.0897	0.1654	**0.0012**	–	**0.0024**	0.4017	0.0566	**0.0490**	**0.0028**	0.9994
TNF-IR/INT combination study	**0.0453**	0.2511	0.8391	0.0866	–	**0.0379**	0.9770	**0.0071**	0.5114	0.0929	–
Monotherapy study	**0.0028**	0.9442	0.1778	0.1462	0.4771	0.4249	0.5984	0.5878	**0.0316**	**0.0337**	–
Baseline CRP											
MTX-IR combination study	0.9068	0.5979	0.6065	0.0537	–	0.2589	0.4199	0.3974	0.3455	0.2497	**0.0221**
TNF-IR/INT combination study	0.0948	0.3845	0.6999	0.8008	–	0.8900	0.4386	0.1774	**0.0159**	0.6836	–
Monotherapy study	**0.0314**	0.0759	**0.0101**	**0.0074**	**0.0055**	**0.0152**	0.5015	0.1328	0.0570	**0.0006**	–
Baseline ESR											
Monotherapy study	0.5073	0.8452	0.9318	0.7010	0.1295	0.5070	**0.0123**	0.9278	0.2552	0.6488	–
Baseline SDAI											
MTX-IR combination study	0.5963	0.5269	0.4816	0.1452	–	**0.0399**	0.9009	0.7552	0.6002	0.5804	0.7226
TNF-IR/INT combination study	0.5986	0.9399	0.5674	NC	–	NC	0.4333	0.1970	0.2160	NC	–
Prior bDMARD											
MTX-IR combination study	0.8583	0.5175	0.1973	0.9345	–	0.8793	0.2421	0.2202	0.8858	0.6812	0.2246
Prior anti-TNFs											
TNF-IR/INT combination study	0.1215	0.2340	0.0925	0.2052	–	0.2210	0.1916	0.2797	0.2853	0.3850	–
Prior csDMARDs											
MTX-IR combination study	0.4208	0.4886	0.6909	0.3946	–	0.1183	0.8905	0.2846	0.9064	0.9111	0.3586
TNF-IR/INT combination study	0.4941	0.6441	0.3744	0.3431	–	0.1108	0.6155	0.8227	0.2881	0.7416	–
Monotherapy study	0.7935	0.2370	0.6967	0.8694	0.3740	0.5514	0.5602	0.5459	0.3767	0.9655	–
Background csDMARDs											
TNF-IR/INT combination study	0.3194	0.6946	0.3646	0.1658	–	0.5573	0.2718	0.3391	0.5595	0.8590	–
MTX history											
Monotherapy study	0.5540	0.3982	0.3313	0.2163	0.2163	0.2583	0.6631	0.1498	0.3692	0.6913	–
Glucocorticoid use											
MTX-IR combination study	0.5072	0.6390	0.8555	0.3366	–	0.3784	0.7197	0.8891	0.1751	0.7058	0.9422
TNF-IR/INT combination study	0.2751	0.9400	0.8334	0.6142	–	0.6061	0.0656	0.5640	0.0994	0.8873	–
Monotherapy study	0.0907	0.1650	0.9422	0.0663	**0.0414**	**0.0195**	0.4352	**0.0025**	0.0962	0.7536	–

*ACPA* anti-cyclic citrullinated peptide antibody, *ACR20/50/70* American College of Rheumatology 20%/50%/70% response, *bDMARD *biological and targeted disease-modifying antirheumatic drug, *BMI* body mass index, *CDAI* Clinical Disease Activity Index, *CRP* C-reactive protein, *csDMARD* conventional synthetic disease-modifying antirheumatic drug, *DAS28-CRP* Disease Activity Score in 28 joints using CRP, *DAS28-ESR* Disease Activity Score in 28 joints using erythrocyte sedimentation rate, *HAQ-DI* Health Assessment Questionnaire-Disability Index, *INT* intolerant, *IR* inadequate response, *NC* not calculated, *mTSS* Modified Total Sharp Score, *MTX* methotrexate, *RA* rheumatoid arthritis, *RF* rheumatoid factor, *SDAI* Simplified Disease Activity Index, *TNF* tumour necrosis factor

*Assessed at week 16/12/24 in the MTX-IR combination study, TNF-IR/INT combination study, and monotherapy studies, respectively

**Assessed at week 52

#### Age and sex

No treatment-by-subgroup interaction *p* values < 0.05 were found for subpopulations defined by age or sex for ACR responses, CDAI-based endpoints, and DAS28-based endpoints (Table 1). In the MTX-IR combination study, there were also no interaction *p* values < 0.05 for age or sex and change from baseline in mTSS (Table 1). In the TNF-IR/INT combination study, the 95% CI was wide and crossed 0 for change from baseline in HAQ-DI at week 12 in male patients (Figure S5 (Additional file 1); Table 1). In each of the three studies, ORs or LSM treatment differences favoured both doses of sarilumab combination therapy over placebo + csDMARDs or sarilumab monotherapy over adalimumab across the majority of endpoints independent of age or sex (Figs. 1, 2, and 3 and Figures S1, S3, S6 (Additional file 1)).

#### Race and region

No treatment-by-subgroup interaction *p* values < 0.05 were reported for any endpoints for race or region for the MTX-IR combination study and the monotherapy study (Table 1). ORs and LSM treatment differences consistently favoured sarilumab treatment over placebo or adalimumab across the majority of endpoints independent of race or region (Fig. 2, Figures S1, S3, S4, S6 (Additional file 1)). In the TNF-IR/INT combination study, ORs and LSM treatment differences consistently favoured sarilumab + csDMARDs over placebo + csDMARDs on all efficacy endpoints assessed for subgroups defined by race or region except for CDAI remission for non-white patients (i.e. race ‘others’; *n* = 158). However, the 95% CI for this point estimate was wide and crossed 0 (Figure S4B (Additional file 1)). An interaction test with a nominal *p* < 0.05 was found between race and ACR20 and ACR50 response (*p* = 0.03 and *p* = 0.02) and CDAI remission (*p* < 0.01), but not for ACR70 and CDAI improvement ≥ 58% (Table 1).

#### Weight and BMI

LSM treatment differences generally favoured sarilumab (± csDMARDs) over placebo + csDMARDs or adalimumab, irrespective of weight category for change from baseline in DAS28-CRP (Fig. 2), CDAI (Figure S3 (Additional file 1)), and mTSS (assessed only in the MTX-IR combination study, Figure S6 (Additional file 1)). ORs also favoured sarilumab over placebo + csDMARDs for CDAI remission irrespective of weight category (Figure S4 (Additional file 1)), and treatment-by-subgroup interaction *p* values were ≥ 0.05 for all endpoints (Table 1). Altogether, 95% CIs for weight categories were wide and overlapping for all endpoints.

For BMI, interaction-by-subgroup *p* values were ≥ 0.05 for the two combination studies (with the exception of DAS28-CRP in the MTX-IR combination study). ORs and LSM treatment differences favoured sarilumab 150/200 mg over placebo + MTX regardless of BMI subgroup in the MTX-IR and TNF-IR/INT combination studies across all efficacy endpoints. In the monotherapy study, however, interactions with nominal *p* < 0.05 (indicating a difference in sarilumab efficacy) were found for BMI across most efficacy endpoints compared to adalimumab (Table 1). BMI < 25 kg/m^2^ and ≥ 25 to < 30 kg/m^2^ was associated with more robust sarilumab responses than BMI ≥ 30 kg/m^2^ for several efficacy endpoints, including those based on ACR, CDAI, and HAQ-DI.

#### Baseline autoantibody status

Across the three studies, treatment-by-subgroup interaction values *p* < 0.05 (indicating an effect of baseline ACPA status) were consistently observed for ACR20 (Table 1). Compared with patients who were ACPA negative at baseline, LSM treatment differences and ORs consistently demonstrated a numerically greater treatment effect for most endpoints with sarilumab (± csDMARDs) for patients who were ACPA positive at baseline, particularly with the 200 mg sarilumab dose (sarilumab 200 mg; *n* = 337 and *n* = 137 in the MTX-IR and TNF-IR/INT combination studies, respectively).

Treatment-by-subgroup interaction *p* values < 0.05 were not consistently observed for any endpoint across the three studies for RF status at baseline. Among RF-negative patients in the MTX-IR combination study (200 mg group, *n* = 69), although ORs and LSMs appeared to favour 200 mg sarilumab + MTX for some endpoints, treatment-by-subgroup *p* values were all ≥ 0.05 (suggesting sarilumab efficacy may not be impacted overall by RF status). Similarly, among RF-negative patients in the TNF-IR/INT combination study, ORs and LSMs appeared to favour 200 mg sarilumab + csDMARDs for some endpoints; however, treatment interactions for RF status had nominal *p* < 0.05 only for ACR20/50, CDAI ≥ 58%, and change from baseline in HAQ-DI. In the monotherapy study, the only significant treatment interaction *p* value for RF status was for ACR20 (Table 1).

### Safety

As reported previously, across the three sarilumab RCTs and in a large, long-term safety analysis, the most frequent AEs were neutropenia, increased alanine aminotransferase (ALT), injection site erythema, upper respiratory infections, urinary tract infections, nasopharyngitis, and bronchitis [17–19, 24].

Due to the particular concern for the safety and tolerability of treatments in patients aged ≥ 65 years, we assessed AEs in this population. TEAEs and SAEs occurred more frequently in the small group of patients aged ≥ 65 years (*n* = 289) compared with patients aged < 65 years (*n* = 1819) for the placebo, sarilumab, and adalimumab treatment groups (Table 2). Infections were the most common TEAEs in both age groups treated with sarilumab, but serious infections were uncommon (Table 2).

**Table 2:** Overview of TEAEs by age

*n*/*N* (%)	Combination therapy with MTX in MTX-IR patients 52 weeks			Combination therapy with csDMARDs in TNF-IR/INT patients 24 weeks			Monotherapy in MTX-IR/INT patients 24 weeks	
	Placebo	Sarilumab 150 mg q2w	Sarilumab 200 mg q2w	Placebo	Sarilumab 150 mg q2w	Sarilumab 200 mg q2w	Adalimumab 40 mg q2w	Sarilumab 200 mg q2w
TEAEs								
Patients aged < 65 years	217/356 (61.0)	268/360 (74.4)	267/345 (77.4)	76/152 (50.0)	99/150 (66.0)	102/154 (66.2)	105/144 (72.9)	113/158 (71.5)
Patients aged ≥ 65 years	29/41 (70.7)	32/41 (78.0)	45/51 (88.2)	14/29 (48.3)	20/31 (64.5)	18/30 (60.0)	36/40 (90.0)	14/26 (53.8)
Serious AEs								
Patients aged < 65 years	17/356 (4.8)	31/360 (8.6)	33/345 (9.6)	4/152 (2.6)	6/150 (4.0)	6/154 (3.9)	7/144 (4.9)	7/158 (4.4)
Patients aged ≥ 65 years	4/41 (9.8)	6/41 (14.6)	12/51 (23.5)	2/29 (6.9)	0/31 (0.0)	4/30 (13.3)	11/40 (27.5)	3/26 (11.5)
AE leading to death								
Patients aged < 65 years	2/356 (0.6)	1/360 (0.3)	1/345 (0.3)	1/152 (0.7)	0/150 (0.0)	0/154 (0.0)	1/144 (0.7)	1/158 (0.6)
Patients aged ≥ 65 years	0/41 (0.0)	1/41 (2.4)	0/51 (0.0)	0/29 (0.0)	0/31 (0.0)	0/30 (0.0)	0/40 (0.0)	0/26 (0.0)
AE leading to discontinuation								
Patients aged < 65 years	17/356 (4.8)	44/360 (12.2)	42/345 (12.2)	6/152 (3.9)	11/150 (7.3)	13/154 (8.4)	10/144 (6.9)	10/158 (6.3)
Patients aged ≥ 65 years	5/41 (12.2)	8/41 (19.5)	12/51 (23.5)	2/29 (6.9)	3/31 (9.7)	4/30 (13.3)	4/40 (10.0)	3/26 (11.5)
Infections								
Patients aged < 65 years	112/356 (31.5)	149/360 (41.4)	133/345 (38.6)	43/152 (28.3)	35/150 (23.3)	45/154 (29.2)	49/144 (34.0)	54/158 (34.2)
Patients aged ≥ 65 years	15/41 (36.6)	19/41 (46.3)	26/51 (51.0)	5/29 (17.2)	5/31 (16.1)	11/30 (36.7)	15/40 (37.5)	4/26 (15.4)
Serious infections								
Patients aged < 65 years	9/356 (2.5)	7/360 (1.9)	13/345 (3.8)	1/152 (0.7)	1/150 (0.7)	2/154 (1.3)	2/144 (1.4)	2/158 (1.3)
Patients aged ≥ 65 years	1/41 (2.4)	4/41 (9.8)	2/51 (3.9)	1/29 (3.4)	0/31 (0.0)	0/30 (0.0)	1/40 (2.5)	0/26 (0.0)
Opportunistic infections								
Patients aged < 65 years	2/356 (0.6)	2/360 (0.6)	3/345 (0.9)	1/152 (0.7)	0/150 (0.0)	2/154 (1.3)	0/144 (0.0)	1/158 (0.6)
Patients aged ≥ 65 years	0/41 (0.0)	0/41 (0.0)	1/51 (2.0)	0/29 (0.0)	0/31 (0.0)	0/30 (0.0)	1/40 (2.5)	0/26 (0.0)
Tuberculosis								
Patients aged < 65 years	0/356 (0.0)	0/360 (0.0)	0/345 (0.0)	0/152 (0.0)	0/150 (0.0)	0/154 (0.0)	0/26 (0.0)	1/158 (0.6)
Patients aged ≥ 65 years	0/41 (0.0)	0/41 (0.0)	0/51 (0.0)	0/29 (0.0)	0/31 (0.0)	0/30 (0.0)	1/40 (2.5)	0/26 (0.0)
ANC < 1.5 × 10^9^/L*								
Patients aged < 65 years	5/356 (1.4)	66/360 (18.3)	81/345 (23.5)	1/152 (0.7)	24/150 (16.0)	32/154 (20.8)	31/144 (21.5)	52/158 (32.9)
Patients aged ≥ 65 years	1/41 (2.4)	7/41 (17.1)	12/51 (23.5)	1/29 (3.4)	7/31 (22.6)	10/30 (33.3)	9/40 (22.5)	10/26 (38.5)
ANC ≥ 0.5–1 × 10^9^/L (grade 3)								
Patients aged < 65 years	0 (0.0)	16 (4.4)	26 (7.5)	0 (0.0)	9 (6.0)	11 (7.1)	15 (10.4)	23 (14.6)
Patients aged ≥ 65 years	0 (0.0)	3 (7.3)	3 (5.9)	1 (3.4)	1 (3.2)	5 (16.7)	3 (7.5)	4 (15.4)
ANC < 0.5 × 10^9^/L (grade 4)								
Patients aged < 65 years	0 (0.0)	4 (1.1)	1 (0.3)	0 (0.0)	2 (1.3)	1 (0.6)	0 (0.0)	0 (0.0)
Patients aged ≥ 65 years	0 (0.0)	0 (0.0)	1 (2.0)	0 (0.0)	2 (6.5)	1 (3.3)	0 (0.0)	3 (11.5)
Platelets < 50 × 10^9^/L								
Patients aged < 65 years	0/356 (0.0)	0/360 (0.0)	2/345 (0.6)	0/152 (0.0)	0/150 (0.0)	1/154 (0.6)	0/144 (0.0)	1/158 (0.6)
Patients aged ≥ 65 years	0/41 (0.0)	0/41 (0.0)	0/51 (0.0)	0/29 (0.0)	0/31 (0.0)	0/30 (0.0)	0/40 (0.0)	0/26 (0.0)
ALT > 3 to ≤ 5× ULN								
Patients aged < 65 years	7/356 (2.0)	24/360 (6.7)	24/345 (7.0)	2/152 (1.3)	4/150 (2.7)	6/154 (3.9)	8/144 (5.6)	9/158 (5.7)
Patients aged ≥ 65 years	1/41 (2.4)	1/41 (2.4)	0/51 (0.0)	0/29 (0.0)	0/31 (0.0)	1/30 (3.3)	1/40 (2.5)	0/26 (0.0)
ALT > 5 to ≤ 10× ULN								
Patients aged < 65 years	1/356 (0.3)	9/360 (2.5)	9/345 (2.6)	0/152 (0.0)	0/150 (0.0)	0/154 (0.0)	2/144 (1.4)	3/158 (1.9)
Patients aged ≥ 65 years	0/41 (0.0)	0/41 (0.0)	0/51 (0.0)	0/29 (0.0)	0/31 (0.0)	0/30 (0.0)	0/40 (0.0)	0/26 (0.0)

*AE* adverse event, *ALT* alanine aminotransferase, *ANC* absolute neutrophil count, *csDMARD* conventional synthetic disease-modifying antirheumatic drug, *INT* intolerant, *IR* inadequate response, *MTX* methotrexate, *q2w* every 2 weeks, *TNF* tumour necrosis factor

*For Black patients, a cut-off value of < 1.0 × 10^9^ ANC is reported, due to differences in ANC reference ranges according to racial group

In general, no appreciable differences were observed between subpopulations ≥ 65 and < 65 years for laboratory parameters, such as ANC, platelets, and ALT. In the MTX-IR combination study and monotherapy study, the proportion of patients with ANC < 1.5 × 10^9^/L was comparable in both age groups across all treatment groups. In the TNF-IR/INT combination study, compared with patients aged < 65 years, a higher proportion of patients aged ≥ 65 years had ANC < 1.5 × 10^9^/L across placebo and sarilumab treatment groups: 3.4% (*n* = 1/29) vs. 0.7% (*n* = 1/152) with placebo + csDMARDs, 22.6% (*n* = 7/31) vs. 16.0% (*n* = 24/150) with sarilumab 150 mg q2w + csDMARDs, and 33.3% (*n* = 10/30) vs. 20.8% (*n* = 32/154) with sarilumab 200 mg q2w + csDMARDs (Table 2). Proportions of patients with platelets < 50 × 10^9^/L were low in all age/treatment groups (≤ 0.6%).

## Discussion

These subanalyses of three separate, phase III RCTs, which included patients with active RA, demonstrated that sarilumab consistently improved signs and symptoms of RA, physical functioning, and radiographic progression across a wide range of patient subpopulations, representing a broad range of patients.

Long-term safety data for sarilumab treatment have previously been reported [24]. Our analysis here was consistent with previous analyses of safety data in the pooled population, which showed no clear differences in the safety profile of sarilumab between patient subgroups, although a slightly higher proportion of patients aged ≥ 65–< 75 years reported serious infections in the sarilumab 150 mg q2w + csDMARD group vs. those aged ≥ 75 years: 4/79 (5.1%) vs. 8/574 (1.4%) in the placebo-controlled safety population [25].

In the present analysis, whilst the patient population aged ≥ 65 years was small, limiting interpretation of these data, higher rates of SAEs, and serious infections were observed in these patients compared to those aged < 65 years; however, no appreciable differences were observed between patients aged < 65 vs. ≥ 65 years for ANC, ALT, and platelet count. Safety and tolerability are a specific potential concern in this subpopulation, and AEs are among the main causes of discontinuation of RA therapy among older patients [26]. An increase in comorbidities, such as diabetes mellitus and renal disease, in older patients vs. younger patients or more frequent use of oral glucocorticoids has been suggested to increase the risk of infections [27–29]. Previous studies have also reported an increased risk of serious infections in RA patients > 60 years of age compared with younger patients [30–32], but no significant difference was found in the rate of infection between the ages of 65–74 and ≥ 75 years [30].

In interpreting our findings, it is critical to understand the appropriate statistical methodology for assessing whether treatment effects vary across levels of a baseline variable/characteristic. A common error is to conduct separate tests of treatment effects within each of the levels of the baseline variable or to evaluate the observed treatment effect sizes within each subgroup [33]; the correct approach is to conduct a statistical test for interaction [33]. An interaction test with nominal *p* < 0.05 suggests that the baseline variable has an impact on the treatment effect and, in itself, does not indicate how the effect of treatment differs across the baseline characteristic. In our analysis, the interaction test also does not differentiate between the two sarilumab doses evaluated in the placebo-controlled studies; furthermore, it was not the aim of this analysis to compare the two approved doses. Taking a conservative approach, in our analysis, we have noted the subgroups for which the interaction test had a nominal *p* < 0.05; however, it has been suggested that a preferable way of assessing the *p* value is that as it gets smaller, the subgroup hypothesis becomes increasingly credible and should only be taken seriously when *p* values reach 0.001 or less [34]. Additionally, we should appreciate that the probability of a false-positive finding increases when multiple subgroup analyses are performed and that criteria to assess the credibility of subgroup analyses should include whether the interaction is consistent across studies and consistent across closely related outcomes within the study [34]. In our analysis, interaction tests with nominal *p* < 0.05 that were consistent across the studies were only seen for baseline ACPA status for ACR20 (but not ACR50 nor ACR70) and for no other baseline variable evaluated.

ACPA-positive and RF-positive RA are associated with worse prognoses and erosive disease [7–9]. Acknowledging the reliability criteria above, it was interesting to note interaction *p* values were < 0.05 for ACR20 (but not for ACR50 and ACR70), with forest plots indicating sarilumab may be more effective in RA patients who are ACPA positive. An effect of autoantibody status on efficacy has been observed for other biologic treatments; ACPA-positive status at baseline was associated with a superior response to both adalimumab and abatacept in the AMPLE study [11]. Furthermore, responses to abatacept were greater in those patients with high titres of ACPA at baseline, although this association was not observed for adalimumab [11]. Responses to rituximab as well as csDMARDs ± glucocorticoids have also been reported to differ by ACPA status [14, 35]. In contrast, analyses of IL-6 receptor (IL-6R) inhibition with tocilizumab have not identified a relationship between baseline ACPA or RF status and achievement of response and remission [36, 37]. In our analysis, treatment interaction *p* values for RF status were not < 0.05 consistently across studies; indeed, *p* values ≥ 0.05 were observed for all endpoints in the largest of the three studies. As seronegative patients may have less aggressive disease, it may be more difficult to determine a treatment difference in these patients. Additionally, based on the inclusion/exclusion criteria for the three pivotal phase III sarilumab studies, there were fewer seronegative patients enrolled, and the smaller sample size means that these data may be less reliable.

In the analysis presented here, we found no effect of baseline weight on sarilumab efficacy. There was also no indication that baseline BMI impacts the efficacy of sarilumab + csDMARDs. In the monotherapy study, potential interactions with baseline BMI were identified; however, this observation and the lack of interaction with baseline weight in the monotherapy study are inconsistent.

Although no consistent treatment-by-subgroup interactions were seen for baseline CRP in the combination studies, potential interactions were identified for baseline CRP for some endpoints assessed in the monotherapy trial, with a greater magnitude of effect in patients with CRP > 15 mg/L at baseline. These interactions were not observed in the related subgroup categorized by baseline erythrocyte sedimentation rate (ESR), and the efficacy results in the subgroups categorized by baseline CRP were consistent with the main results. Change in CRP levels in the initial weeks following treatment initiation has been reported to predict response to TNF inhibitors [38, 39]. Our findings are also consistent with some studies of IL-6R inhibition with tocilizumab, which have reported a greater EULAR response in patients with high baseline CRP [37].

The different inclusion criteria of the three studies have already provided some insight that sarilumab is effective in patients with RA irrespective of prior therapies [17–19]. Our analysis provides more information on the effects of prior treatments with csDMARDs (number of prior agents and INT or IR to MTX) and bDMARDs (including whether patients had received one or more prior treatment) and found no differences in the efficacy of sarilumab across endpoints. We also evaluated the effect of stable glucocorticoid use and observed no treatment-by-subgroup interaction for sarilumab + csDMARDs. Interactions for baseline glucocorticoid use were not seen consistently across endpoints in the monotherapy study.

A limitation of the present analysis is that the study populations recruited in the three individual trials differed temporally, spatially (i.e. by geographical location), and by prior treatment history and response to previous treatment; thus, results across the studies cannot be compared directly. In addition, there were some differences between the prespecified subpopulations. The duration of the studies and small numbers of patients in some subpopulations also limit the interpretation of the data. The inherent limitations of conducting multiple subgroup analyses should also be noted, with the probability of a false-positive finding increasing with the number of analyses performed [33]; indeed, several of the treatment-by-subgroup interactions identified in the current analysis were not replicated on related endpoints. However, within similar backgrounds and sarilumab regimens, some trends were noted. It is likely that treatment responses in patients with RA are influenced by a combination of baseline characteristics rather than isolated characteristics, and additional analyses with larger subpopulations are warranted to investigate this further.

## Conclusions

In patients with RA and MTX-IR/INT or TNF-IR/INT, sarilumab (± csDMARDs) demonstrated superiority to placebo (± csDMARDs) or adalimumab for clinical efficacy measures across many patient subpopulations. Apart from ACPA status, there were no consistent signals indicating differential effects of sarilumab in any of the subpopulations assessed. A larger treatment effect was observed for sarilumab 200 mg q2w in the MTX-IR and TNF-IR/INT combination studies among patients who were seropositive (RF or ACPA) at baseline and for those with lower BMI (< 30 kg/m^2^) at baseline. These data suggest that treatment with sarilumab is associated with beneficial effects across a broad spectrum of patients who have RA.

## Supplementary information

**Additional file 1.** Supplementary methods and results.

## Data Availability

Qualified researchers may request access to patient-level data and related study documents including clinical study report, study protocol with any amendments, blank case report form, statistical analysis plan, and dataset specifications. Patient-level data will be anonymized and study documents will be redacted to protect the privacy of trial participants. Further details on Sanofi’s data sharing criteria, eligible studies, and process for requesting access can be found at https://www.clinicalstudydatarequest.com.

## Abbreviations

ACPA: Anti-cyclic citrullinated peptide antibody
ACR20/50/70: American College of Rheumatology 20%/50%/70% response
AE: Adverse event
ALT: Alanine aminotransferase
ANC: Absolute neutrophil count
bDMARD: Biological and targeted disease-modifying antirheumatic drug
BMI: Body mass index
CDAI: Clinical Disease Activity Index
CI: Confidence interval
CRP: C-reactive protein
csDMARD: Conventional synthetic disease-modifying antirheumatic drug
DAS28-CRP: Disease Activity Score in 28 joints using C-reactive protein
DAS28-ESR: Disease Activity Score in 28 joints using erythrocyte sedimentation rate
ESR: Erythrocyte sedimentation rate
EULAR: European League Against Rheumatism
LSM: Least-squares mean
HAQ-DI: Health Assessment Questionnaire-Disability Index
HDA: High disease activity
IL-6: Interleukin-6
IL-6R: Interleukin-6 receptor
INT: Intolerant
IR: Inadequate response
mTSS: Modified total Sharp/van der Heijde score
MTX: Methotrexate
*n*: Number of evaluable patients regardless of the treatment group
OR: Odds ratio
q2w: Every 2 weeks
RA: Rheumatoid arthritis
RCT: Randomized controlled study
RF: Rheumatoid factor
SAE: Serious treatment-emergent adverse event
SC: Subcutaneous
SDAI: Simplified Disease Activity Index
TEAE: Treatment-emergent adverse event
TNF: Tumour necrosis factor
ULN: Upper limit of normal
