# Supplementary material for: Efficacy and safety of sarilumab in combination with csDMARDs or as monotherapy in subpopulations of patients with moderately to severely active rheumatoid arthritis in three phase III randomized, controlled studies
Source: Arthritis Res Ther. 2020 Jun 10;22:139. doi: 10.1186/s13075-020-02194-z (PMC7288435; doi:10.1186/s13075-020-02194-z)
Supplement: Supplementary file 1 — Additional file 1. Supplementary methods and results. [file 13075_2020_2194_MOESM1_ESM.pdf]

Supplementary information for:

Efficacy and safety of sarilumab in combination with csDMARDs or as monotherapy in subpopulations of patients with moderately to severely active rheumatoid arthritis in three phase III randomized, controlled studies

Mark C. Genovese, Roy Fleischmann, Alan Kivitz, Eun Bong Lee, Hubert van Hoogstraten, Toshio Kimura, Gregory St John, Erin K. Mangan, Gerd R. Burmester

Supplementary Methods

Patient subpopulations

The baseline characteristics prespecified in the individual trial protocols for efficacy analyses stratified by patient subpopulations were:

- Age: < 65, ≥ 65 years
- Sex: male, female
- Race: White, other
- Region: (1) Western countries, (2) South America, (3) rest of world
- Weight: < 50 kg, ≥ 50–<100 kg, ≥ 100 kg for the MTX-IR combination study; < 60 kg, ≥ 60–<100 kg, ≥ 100 kg for the other two studies
- BMI: < 25 kg/m^2, ≥ 25–<30 kg/m^2, ≥ 30 kg/m^2
- Smoking history: never, former, current for the monotherapy study and yes, no for the other two studies
- Duration of RA: ≤ median, > median and ≤ 3 years, > 3 years
- RF: positive, negative
- Anti-cyclic citrullinated peptide antibody: positive, negative
- CRP: ≤ 15, > 15 mg/L

The following were also prespecified for specific studies:

MTX-IR combination study

- Prior biologic use: yes, no
- Number of prior csDMARDs: 0, 1, 2, ≥ 3

TNF-IR/INT combination study

- Number of prior csDMARDs: 0, 1, 2, ≥ 3
- Number of previous anti-TNFs: 1, ≥ 1
- Background DMARD use: MTX, non-MTX

  monotherapy study

- ESR: ≤ median, > median
- MTX history: IR, INT/inappropriate

Outcomes analysed by the following subpopulations were exploratory analyses performed post hoc for all three studies:

Baseline glucocorticoid use, yes, no; SDAI remission / low / moderate disease activity, high disease activity; duration of RA ≤ 2 years, > 2 years; and ESR < 1.2 × ULN and ≥ 1.2 × ULN (ULN defined here as 28 mm/hour based on protocol inclusion criteria).

Supplementary tables and figures

Supplementary Table S1 Selected baseline demographics and disease characteristics across all three trials

	MTX-IR combination study			TNF-IR/INT combination study			Monotherapy study	
	Placebo	Sarilumab 150 mg q2w	Sarilumab 200 mg q2w	Placebo	Sarilumab 150 mg q2w	Sarilumab 200 mg q2w	Adalimumab 40 mg q2w	Sarilumab 200 mg q2w
Age, mean ± SD, years	50.9 ± 11.2	50.1 ± 11.9	50.8 ± 11.8	51.9 ± 12.4	54.0 ± 11.7	52.9 ± 12.9	53.6 ± 11.9	50.9 ± 12.6
Female, *n* (%)	321 (81)	319 (80)	337 (85)	154 (85)	142 (79)	151 (82)	150 (81)	157 (85)
Duration of RA, mean ± SD, years	9.1 ± 8.1	9.5 ± 8.5	8.6 ± 7.0	12.0 ± 10.0	11.6 ± 8.6	12.7 ± 9.6	6.6 ± 7.8	8.1 ± 8.1
Baseline SDAI, mean ± SD	42.7 ± 12.5	42.6 ± 13.0	42.7 ± 12.8	46.9 ± 13.0	44.9 ± 13.3	47.2 ± 15.0	–	–
Baseline CDAI, ± SD	40.6 ± 12.0	40.5 ± 12.5	40.4 ± 12.3	44.2 ± 12.3	42.5 ± 12.9	44.1 ± 13.9	42.4 ± 12.0	43.6 ± 12.1
HAQ-DI mean ± SD	1.6 ± 0.7	1.6 ± 0.6	1.7 ± 0.6	1.8 ± 0.6	1.7 ± 0.6	1.8 ± 0.6	1.6 ± (0.6)	1.6 ± (0.6)
DAS28-CRP mean ± SD	5.9 ± 0.9	6.0 ± 0.9	6.0 ± 0.9	6.2 ± 0.9	6.1 ± 0.9	6.3 ± 1.0	6.0 ± (0.9)	6.0 ± (0.9)

	MTX-IR combination study			TNF-IR/INT combination study			Monotherapy study	
	Placebo	Sarilumab 150 mg q2w	Sarilumab 200 mg q2w	Placebo	Sarilumab 150 mg q2w	Sarilumab 200 mg q2w	Adalimumab 40 mg q2w	Sarilumab 200 mg q2w
RF positive, *n* (%)	336 (84)	345 (87)	328 (83)	142 (79)	135 (75)	132 (73)	116 (65)	119 (67)
ACPA positive, *n* (%)	340 (85)	359 (90)	337 (85)	150 (83)	135 (75)	137 (76)	138 (77)	134 (75)

ACPA anti-cyclic citrullinated peptide antibody, *CDAI* clinical disease activity index, *DAS28-CRP* Disease Activity Score in 28 joints using C-reactive protein, *HAQ-DI* Health Assessment Questionnaire-Disability Index, *INT* intolerant, *IR* inadequate response, *MTX* methotrexate, *n* number of evaluable patients regardless of treatment group, *q2w* every 2 weeks, *RA* rheumatoid arthritis, *RF* rheumatoid factor, *SD* standard deviation, *SDAI* simplified disease activity index, *TNF* tumour necrosis factor.

Figure S1 Odds ratio (95% CI) for ACR50/70 response at week 24 by subpopulation. (**a**) ACR50 response for sarilumab 150/200 mg q2w + MTX vs. placebo + MTX in MTX-IR patients; (**b**) ACR70 response for sarilumab 150/200 mg q2w + MTX vs. placebo + MTX in MTX-IR patients; (**c**) ACR50 response for sarilumab 150/200 mg q2w + csDMARDs vs. placebo + csDMARDs in TNF-IR/INT patients; (**d**) ACR70 response for sarilumab 150/200 mg q2w + csDMARDs vs. placebo + csDMARDs in TNF-IR/INT patients; (**e**) ACR50 response for sarilumab 200 mg q2w vs. adalimumab 40 mg q2w in MTX-IR/INT patients; (**f**) ACR70 response for sarilumab 200 mg q2w vs. adalimumab 40 mg q2w in MTX-IR/INT patients

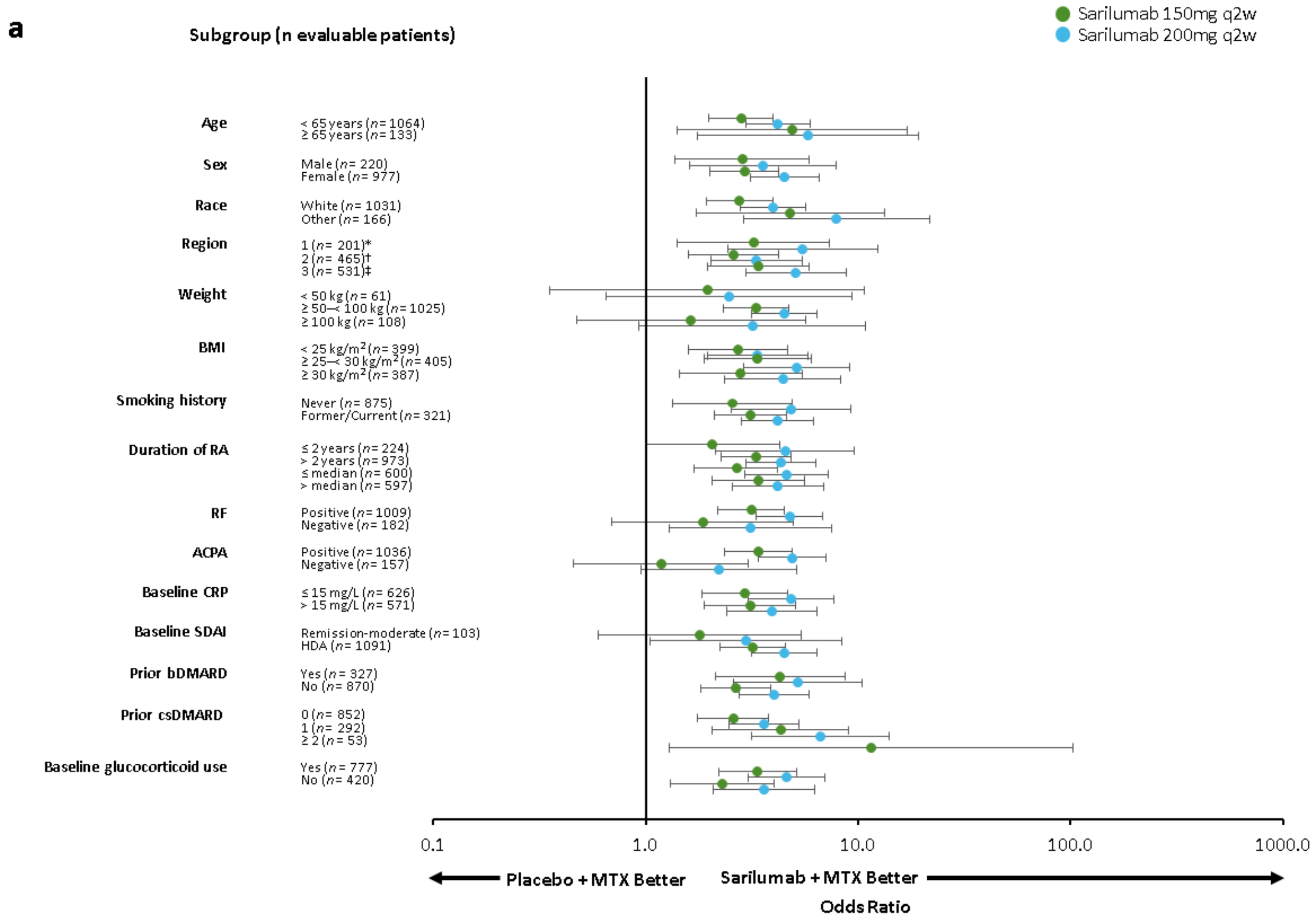

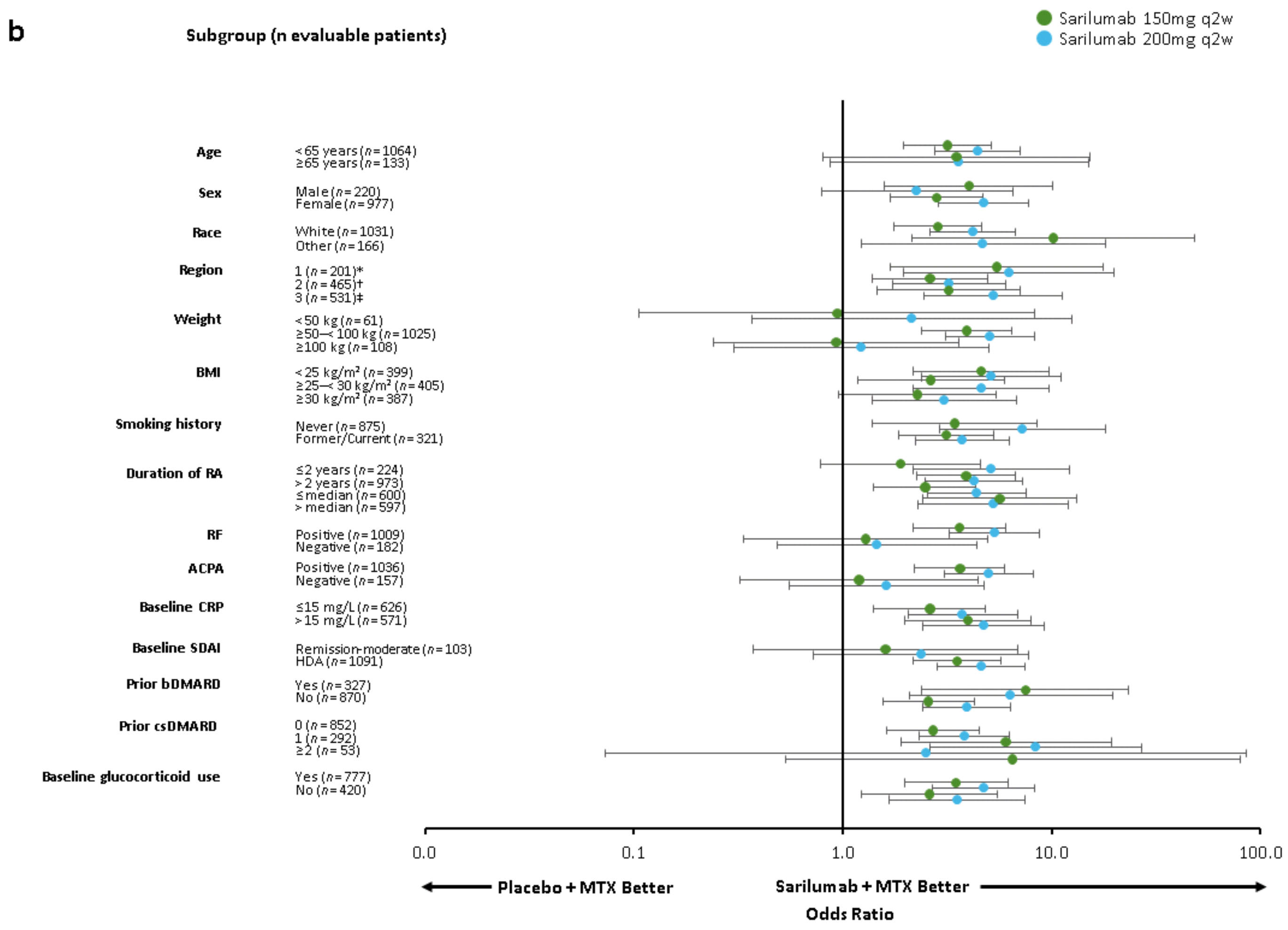
b
Subgroup (n evaluable patients)
Sarilumab 150mg q2w
Sarilumab 200mg q2w
Age
<65 years (n=1064)
≥65 years (n=133)
Sex
Male (n=220)
Female (n=977)
Race
White (n=1031)
Other (n=166)
Region
1 (n=201)*
2 (n=465)†
3 (n=531)‡
Weight
<50 kg (n=61)
≥50–<100 kg (n=1025)
≥100 kg (n=108)
BMI
<25 kg/m² (n=399)
≥25–<30 kg/m² (n=405)
≥30 kg/m² (n=387)
Smoking history
Never (n=875)
Former/Current (n=321)
Duration of RA
≤2 years (n=224)
>2 years (n=973)
≤median (n=600)
>median (n=597)
RF
Positive (n=1009)
Negative (n=182)
ACPA
Positive (n=1036)
Negative (n=157)
Baseline CRP
≤15 mg/L (n=626)
>15 mg/L (n=571)
Baseline SDAI
Remission-moderate (n=103)
HDA (n=1091)
Prior bDMARD
Yes (n=327)
No (n=870)
Prior csDMARD
0 (n=852)
1 (n=292)
≥2 (n=53)
Baseline glucocorticoid use
Yes (n=777)
No (n=420)
0.0
0.1
1.0
10.0
100.0
Placebo + MTX Better
Sarilumab + MTX Better
Odds Ratio

c

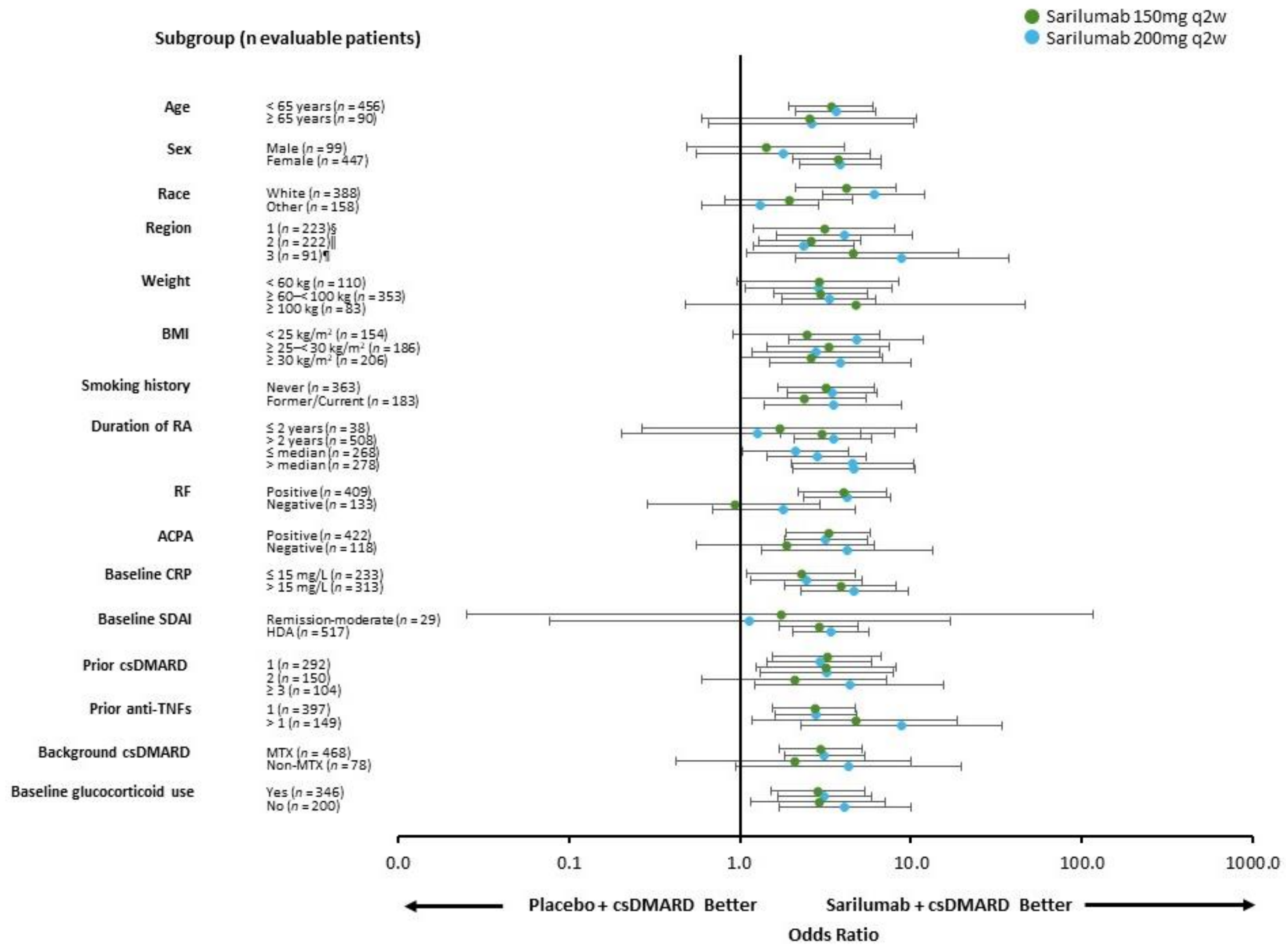
Sarilumab 150mg q2w
Sarilumab 200mg q2w
Subgroup (n evaluable patients)
Age
< 65 years (n = 456)
≥ 65 years (n = 90)
Sex
Male (n = 99)
Female (n = 447)
Race
White (n = 388)
Other (n = 158)
Region
1 (n = 223)§
2 (n = 222)‖
3 (n = 91)¶
Weight
< 60 kg (n = 110)
≥ 60–< 100 kg (n = 353)
≥ 100 kg (n = 83)
BMI
< 25 kg/m² (n = 154)
≥ 25–< 30 kg/m² (n = 186)
≥ 30 kg/m² (n = 206)
Smoking history
Never (n = 363)
Former/Current (n = 183)
Duration of RA
≤ 2 years (n = 38)
> 2 years (n = 508)
≤ median (n = 268)
> median (n = 278)
RF
Positive (n = 409)
Negative (n = 133)
ACPA
Positive (n = 422)
Negative (n = 118)
Baseline CRP
≤ 15 mg/L (n = 233)
> 15 mg/L (n = 313)
Baseline SDAI
Remission-moderate (n = 29)
HDA (n = 517)
Prior csDMARD
1 (n = 292)
2 (n = 150)
≥ 3 (n = 104)
Prior anti-TNFs
1 (n = 397)
> 1 (n = 149)
Background csDMARD
MTX (n = 468)
Non-MTX (n = 78)
Baseline glucocorticoid use
Yes (n = 346)
No (n = 200)
0.0
0.1
1.0
10.0
100.0
1000.0
Placebo + csDMARD Better
Sarilumab + csDMARD Better
Odds Ratio

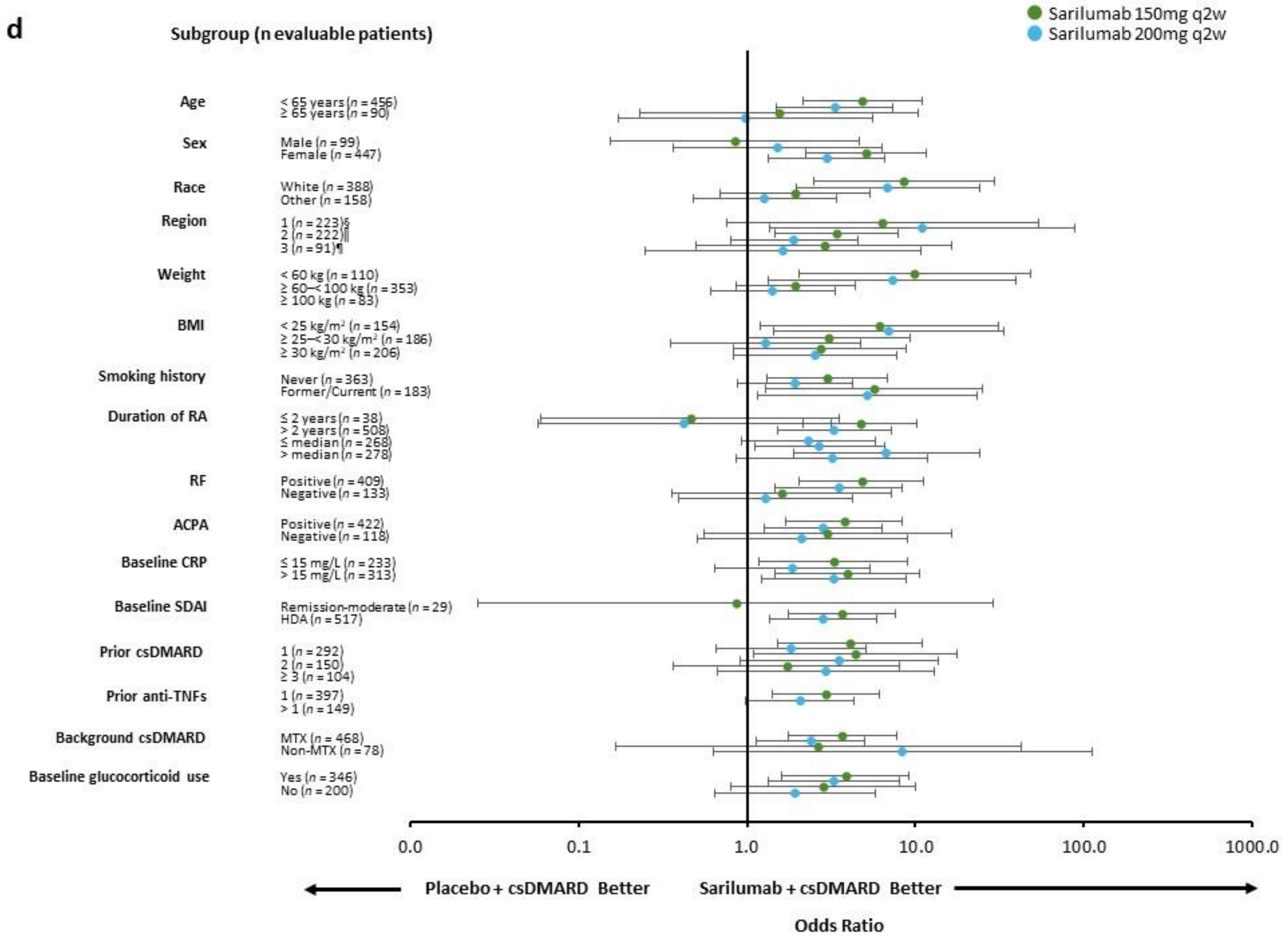
d
Subgroup (n evaluable patients)
Sarilumab 150mg q2w
Sarilumab 200mg q2w
Age
< 65 years (n = 456)
≥ 65 years (n = 90)
Sex
Male (n = 99)
Female (n = 447)
Race
White (n = 388)
Other (n = 158)
Region
1 (n = 223)§
2 (n = 222)‖
3 (n = 91)¶
Weight
< 60 kg (n = 110)
≥ 60–< 100 kg (n = 353)
≥ 100 kg (n = 83)
BMI
< 25 kg/m² (n = 154)
≥ 25–< 30 kg/m² (n = 186)
≥ 30 kg/m² (n = 206)
Smoking history
Never (n = 363)
Former/Current (n = 183)
Duration of RA
≤ 2 years (n = 38)
> 2 years (n = 508)
≤ median (n = 268)
> median (n = 278)
RF
Positive (n = 409)
Negative (n = 133)
ACPA
Positive (n = 422)
Negative (n = 118)
Baseline CRP
≤ 15 mg/L (n = 233)
> 15 mg/L (n = 313)
Baseline SDAI
Remission-moderate (n = 29)
HDA (n = 517)
Prior csDMARD
1 (n = 292)
2 (n = 150)
≥ 3 (n = 104)
Prior anti-TNFs
1 (n = 397)
> 1 (n = 149)
Background csDMARD
MTX (n = 468)
Non-MTX (n = 78)
Baseline glucocorticoid use
Yes (n = 346)
No (n = 200)
0.0
0.1
1.0
10.0
100.0
1000.0
Placebo + csDMARD Better
Sarilumab + csDMARD Better
Odds Ratio

e

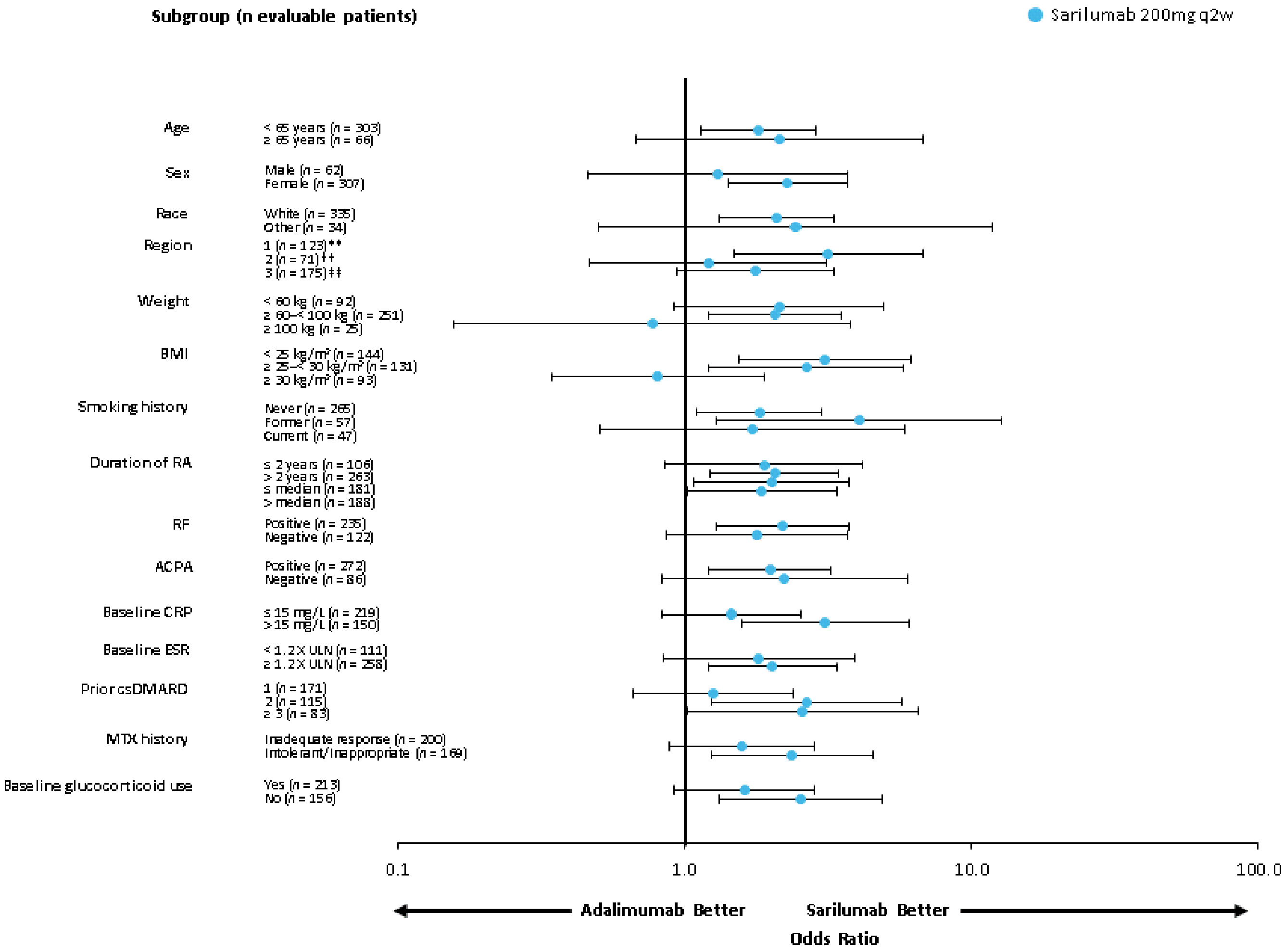
Subgroup (n evaluable patients)
Sarilumab 200mg q2w
Age
< 65 years (n = 303)
≥ 65 years (n = 66)
Sex
Male (n = 62)
Female (n = 307)
Race
White (n = 335)
Other (n = 34)
Region
1 (n = 123)**
2 (n = 71)††
3 (n = 175)‡‡
Weight
< 60 kg (n = 92)
≥ 60–< 100 kg (n = 251)
≥ 100 kg (n = 25)
BMI
< 25 kg/m² (n = 144)
≥ 25–< 30 kg/m² (n = 131)
≥ 30 kg/m² (n = 93)
Smoking history
Never (n = 265)
Former (n = 57)
Current (n = 47)
Duration of RA
≤ 2 years (n = 106)
> 2 years (n = 263)
≤ median (n = 181)
> median (n = 188)
RF
Positive (n = 235)
Negative (n = 122)
ACPA
Positive (n = 272)
Negative (n = 86)
Baseline CRP
≤ 15 mg/L (n = 219)
> 15 mg/L (n = 150)
Baseline ESR
< 1.2X ULN (n = 111)
≥ 1.2X ULN (n = 258)
Prior csDMARD
1 (n = 171)
2 (n = 115)
≥ 3 (n = 83)
MTX history
Inadequate response (n = 200)
Intolerant/Inappropriate (n = 169)
Baseline glucocorticoid use
Yes (n = 213)
No (n = 156)
0.1
1.0
10.0
100.0
Adalimumab Better
Sarilumab Better
Odds Ratio

f

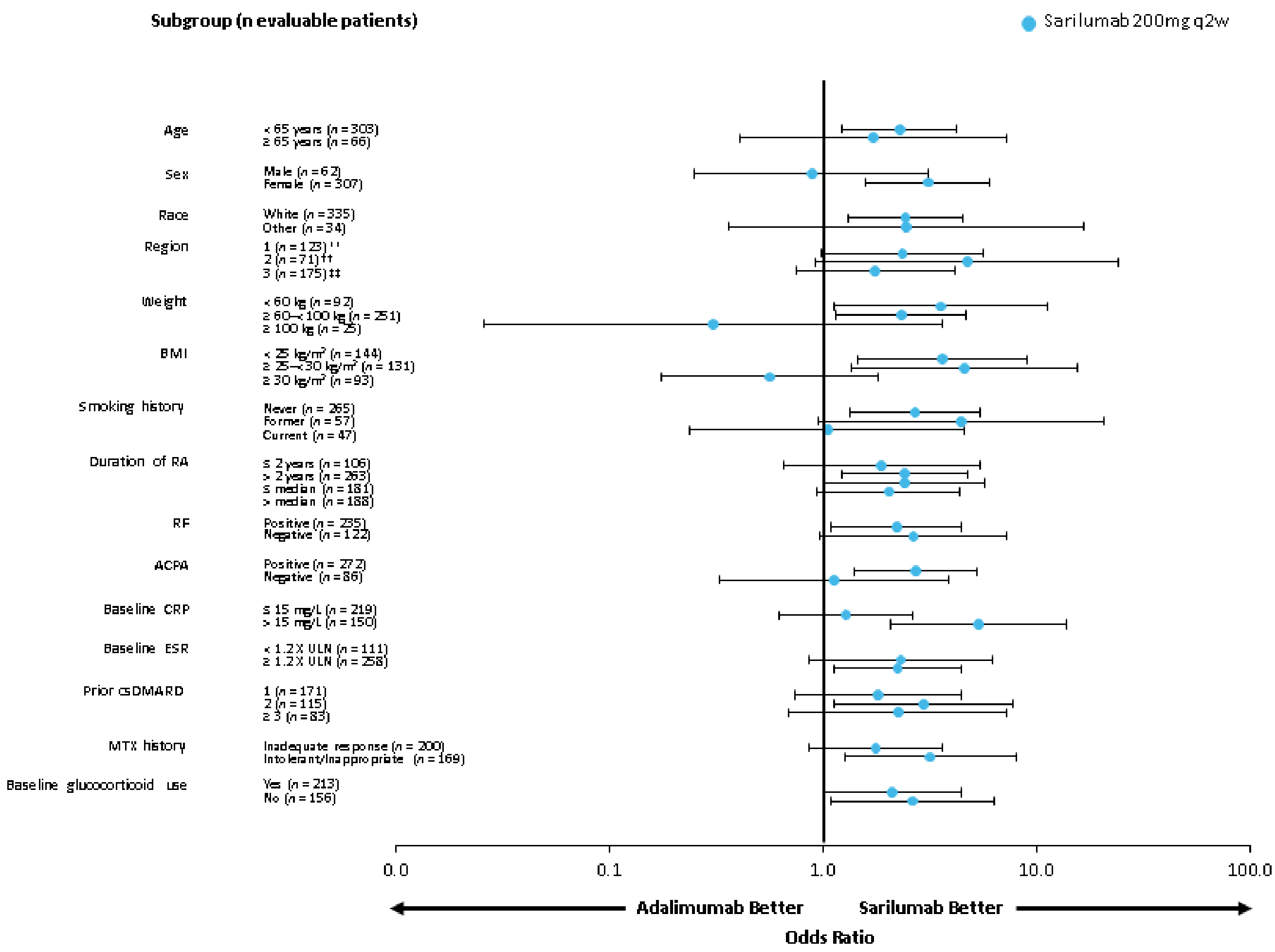

Mantel-Haenszel estimate with terms of (**a,b**) treatment, prior biologic use, region, subpopulation, treatment-by-subpopulation; (**c,d**) treatment, prior anti-TNF use, region, subpopulation, treatment-by-subpopulation; (**e,f**) treatment, region, subpopulation, treatment-by-subpopulation. *ACPA* anti-cyclic citrullinated peptide antibody, *ACR20/50/70* American College of Rheumatology 20%/50%/70% response, *bDMARD* biological and targeted disease-modifying antirheumatic drug, *BMI* body mass index, *CI* confidence interval, *CRP* C-reactive protein, *csDMARD* conventional synthetic disease-modifying antirheumatic drug, *ESR* erythrocyte sedimentation rate, *HDA* high disease activity, *INT* intolerant, *IR* inadequate response, *MTX* methotrexate, *n* number of evaluable patients regardless of treatment group, *q2w* every 2 weeks, *RA* rheumatoid arthritis, *RF* rheumatoid factor, *SDAI* simplified disease activity index, *TNF* tumour necrosis factor, *ULN* upper limit of normal. *Austria, Australia,

Belgium, Canada, Finland, Germany, Greece, Hungary, New Zealand, Norway, Portugal, Spain, and USA; †Argentina, Brazil, Chile, Colombia, and Mexico; ‡Belarus, Estonia, India, Malaysia, Philippines, Poland, Romania, Russia, South Africa, South Korea, Taiwan, Thailand, and Ukraine; §Australia, Canada, Czech Republic, Germany, Greece, Hungary, Israel, Italy, New Zealand, Portugal, Spain, and USA; ‖Argentina, Brazil, Chile, Colombia, Ecuador, Guatemala, Mexico, and Peru; ¶South Korea, Lithuania, Poland, Russia, Taiwan, Turkey, and Ukraine; **Czech Republic, Germany, Hungary, Israel, Spain, and USA; ††Chile and Peru; ‡‡South Korea, Poland, South Africa, Romania, Russia, and Ukraine.

Figure S2. Odds ratio (95% CI) for DAS28-ESR remission (< 2.6) at week 24 by subpopulation for sarilumab 150/200 mg q2w vs. adalimumab in MTX-IR/INT patients

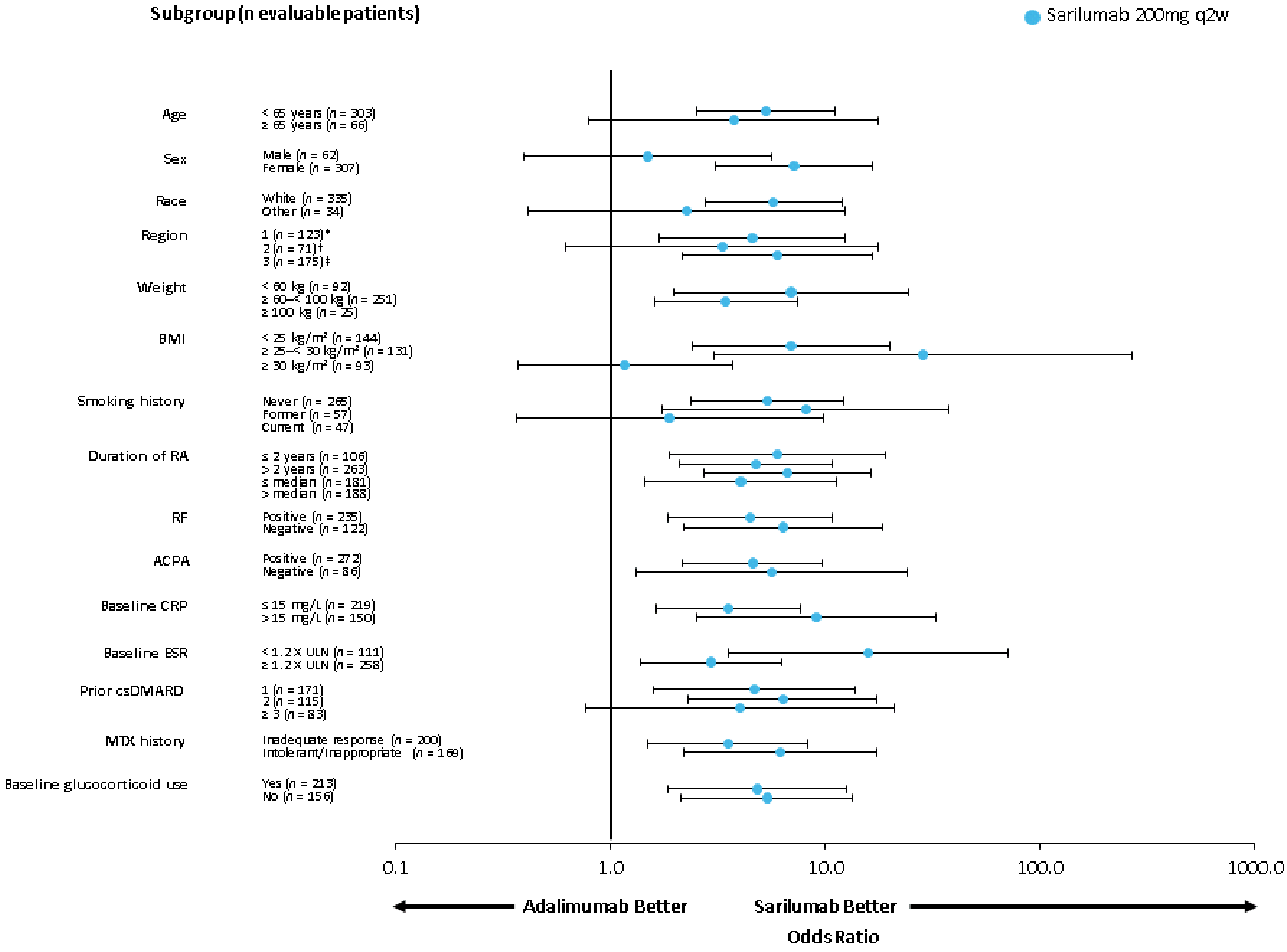

Mantel-Haenszel estimate with model stratified by region. *ACPA* anti-cyclic citrullinated peptide antibody, *BMI* body mass index, *CI* confidence interval, *CRP* C-reactive protein, *csDMARD* conventional synthetic disease-modifying antirheumatic drug, *DAS28-ESR* Disease Activity Score in 28 joints using erythrocyte sedimentation rate, *ESR* erythrocyte sedimentation rate, *INT* intolerant, *IR* inadequate response, *MTX* methotrexate, *n* number of evaluable patients regardless of treatment group, *OR* odds ratio, *q2w* every 2 weeks, *RA* rheumatoid arthritis, *RF* rheumatoid factor, *ULN* upper limit of normal. *Czech Republic, Germany, Hungary, Israel, Spain, and USA; †Chile and Peru; ‡South Korea, Poland, South Africa, Romania, Russia, and Ukraine; §OR not calculated for BMI ≥ 100 kg/m^2.

Figure S3. LSM (95% CI) treatment difference in change from baseline in CDAI at week 24 by subpopulation. (**a**) sarilumab 150/200 mg q2w + MTX vs. placebo + MTX in MTX-IR patients; (**b**) sarilumab 150/200 mg q2w + csDMARDs vs. placebo + csDMARDs in TNF-IR/INT patients; (**c**) sarilumab 200 mg q2w vs. adalimumab 40 mg q2w in MTX-IR/INT patients

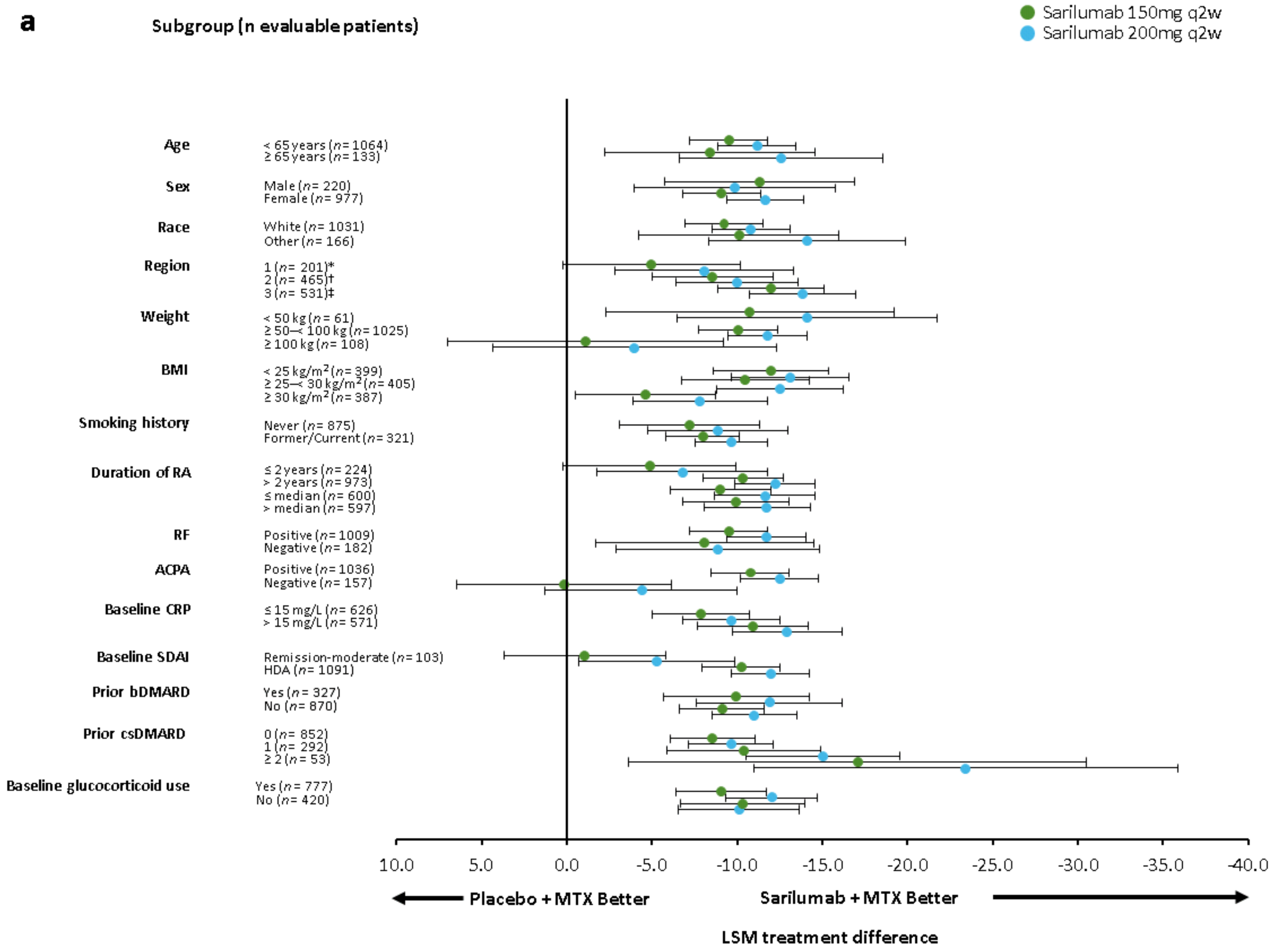

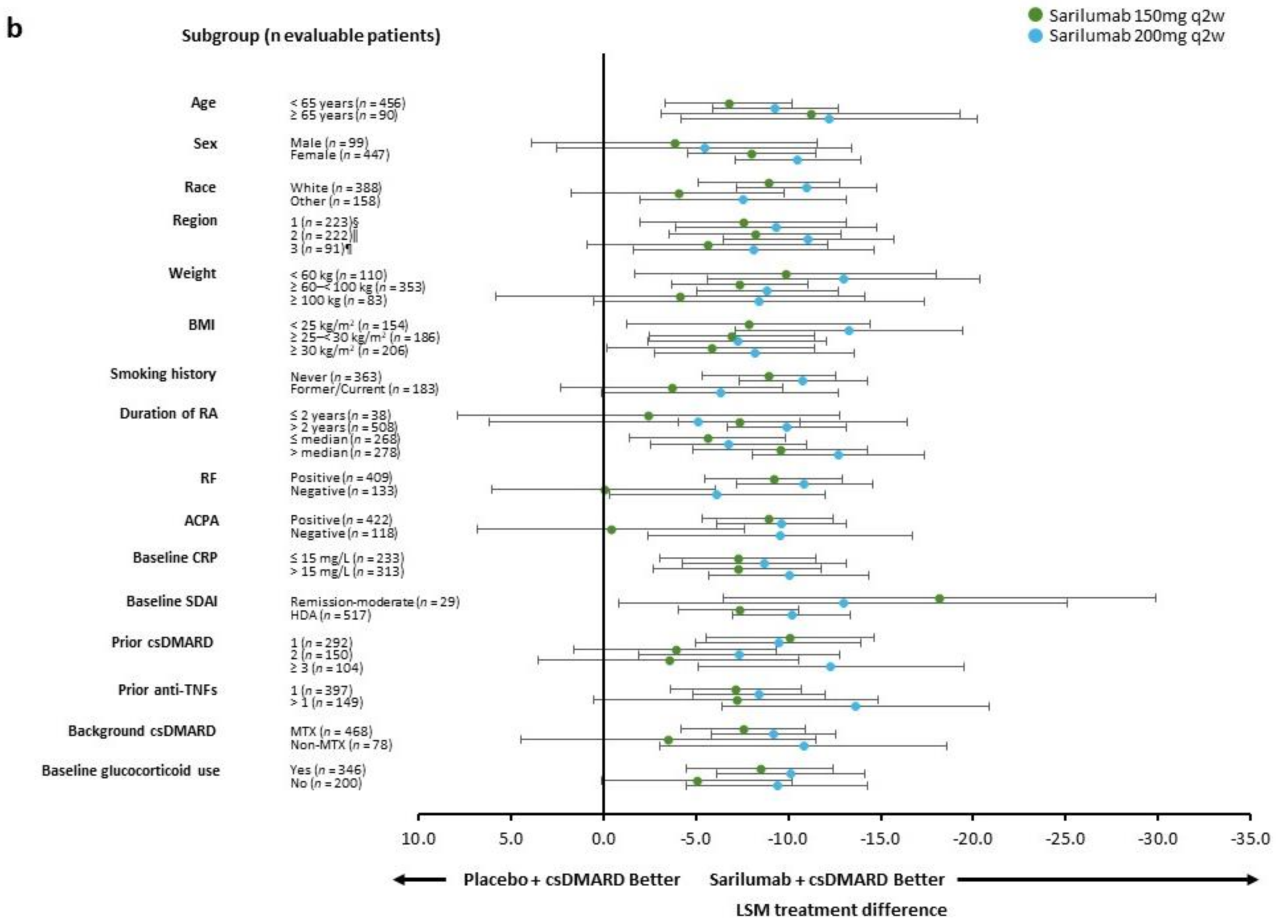
b
Subgroup (n evaluable patients)
Sarilumab 150mg q2w
Sarilumab 200mg q2w
Age
< 65 years (n = 456)
≥ 65 years (n = 90)
Sex
Male (n = 99)
Female (n = 447)
Race
White (n = 388)
Other (n = 158)
Region
1 (n = 223)§
2 (n = 222)||
3 (n = 91)¶
Weight
< 60 kg (n = 110)
≥ 60–< 100 kg (n = 353)
≥ 100 kg (n = 83)
BMI
< 25 kg/m² (n = 154)
≥ 25–< 30 kg/m² (n = 186)
≥ 30 kg/m² (n = 206)
Smoking history
Never (n = 363)
Former/Current (n = 183)
Duration of RA
≤ 2 years (n = 38)
> 2 years (n = 508)
≤ median (n = 268)
> median (n = 278)
RF
Positive (n = 409)
Negative (n = 133)
ACPA
Positive (n = 422)
Negative (n = 118)
Baseline CRP
≤ 15 mg/L (n = 233)
> 15 mg/L (n = 313)
Baseline SDAI
Remission-moderate (n = 29)
HDA (n = 517)
Prior csDMARD
1 (n = 292)
2 (n = 150)
≥ 3 (n = 104)
Prior anti-TNFs
1 (n = 397)
> 1 (n = 149)
Background csDMARD
MTX (n = 468)
Non-MTX (n = 78)
Baseline glucocorticoid use
Yes (n = 346)
No (n = 200)
10.0
5.0
0.0
-5.0
-10.0
-15.0
-20.0
-25.0
-30.0
-35.0
Placebo + csDMARD Better
Sarilumab + csDMARD Better
LSM treatment difference

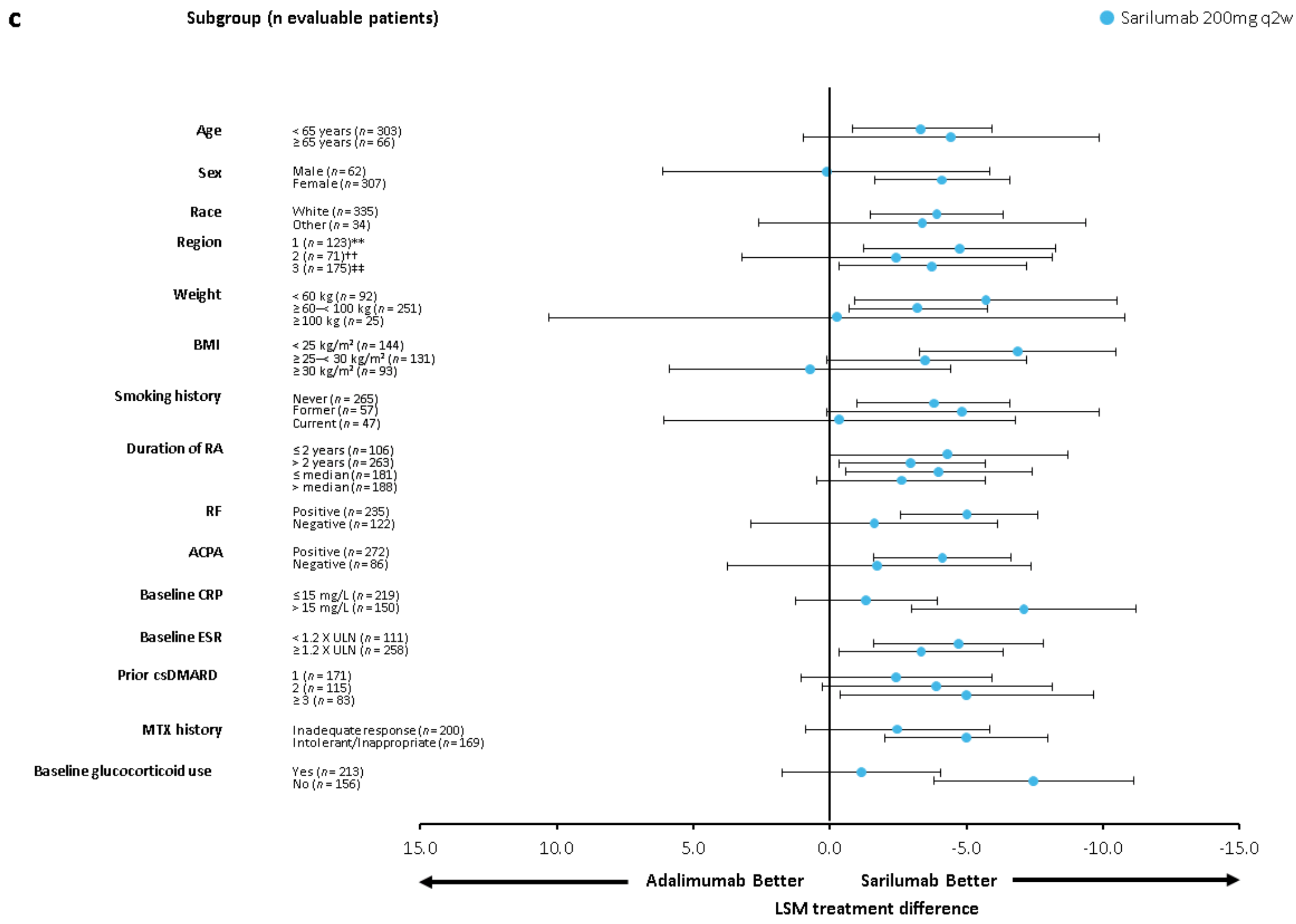

Mixed-effect model for repeated measures with PROC MIXED assuming an unstructured covariance structure: model = (**a**) baseline, treatment, prior bDMARD use, region, visit, treatment-by-interaction; (**b**) baseline, treatment, prior anti-TNF use, region, visit, treatment-by-interaction; (**c**) baseline, treatment, region, visit, treatment-by-interaction. *ACPA* anti-cyclic citrullinated peptide antibody, *bDMARD* biological and targeted disease-modifying antirheumatic drug, *BMI* body mass index, *CDAI* Clinical Disease Activity Index, *CI* confidence interval, *CRP* C-reactive protein, *csDMARD* conventional synthetic disease-modifying antirheumatic drug, *ESR* erythrocyte sedimentation rate, *HDA* high disease activity, *INT* intolerant, *IR* inadequate response, *LSM* least squares mean, *MTX* methotrexate, *n* number of evaluable patients regardless of treatment group, *q2w* every 2 weeks, *RA* rheumatoid arthritis, *RF* rheumatoid factor, *SDAI* simplified disease activity index, *TNF* tumour necrosis factor, *ULN* upper limit of normal. *Austria, Australia, Belgium, Canada,

Finland, Germany, Greece, Hungary, New Zealand, Norway, Portugal, Spain, and USA; †Argentina, Brazil, Chile, Colombia, and Mexico; ‡Belarus, Estonia, India, Malaysia, Philippines, Poland, Romania, Russia, South Africa, South Korea, Taiwan, Thailand, and Ukraine. §Australia, Canada, Czech Republic, Germany, Greece, Hungary, Israel, Italy, New Zealand, Portugal, Spain, and USA; ‖Argentina, Brazil, Chile, Colombia, Ecuador, Guatemala, Mexico, and Peru; ¶South Korea, Lithuania, Poland, Russia, Taiwan, Turkey, and Ukraine. **Czech Republic, Germany, Hungary, Israel, Spain, and USA; ††Chile and Peru; ‡‡South Korea, Poland, South Africa, Romania, Russia, and Ukraine.

Figure S4. Odds ratio (95% CI) for CDAI remission (CDAI ≤ 2.8) at week 24 by subpopulation. (**a**) sarilumab 150/200 mg q2w + MTX vs. placebo + MTX in MTX-IR patients; (**b**) sarilumab 150/200 mg q2w +csDMARDs vs. placebo + csDMARDs in TNF-IR/INT patients; (**c**) sarilumab 200 mg q2w vs. adalimumab 40 mg q2w in MTX-IR/INT patients

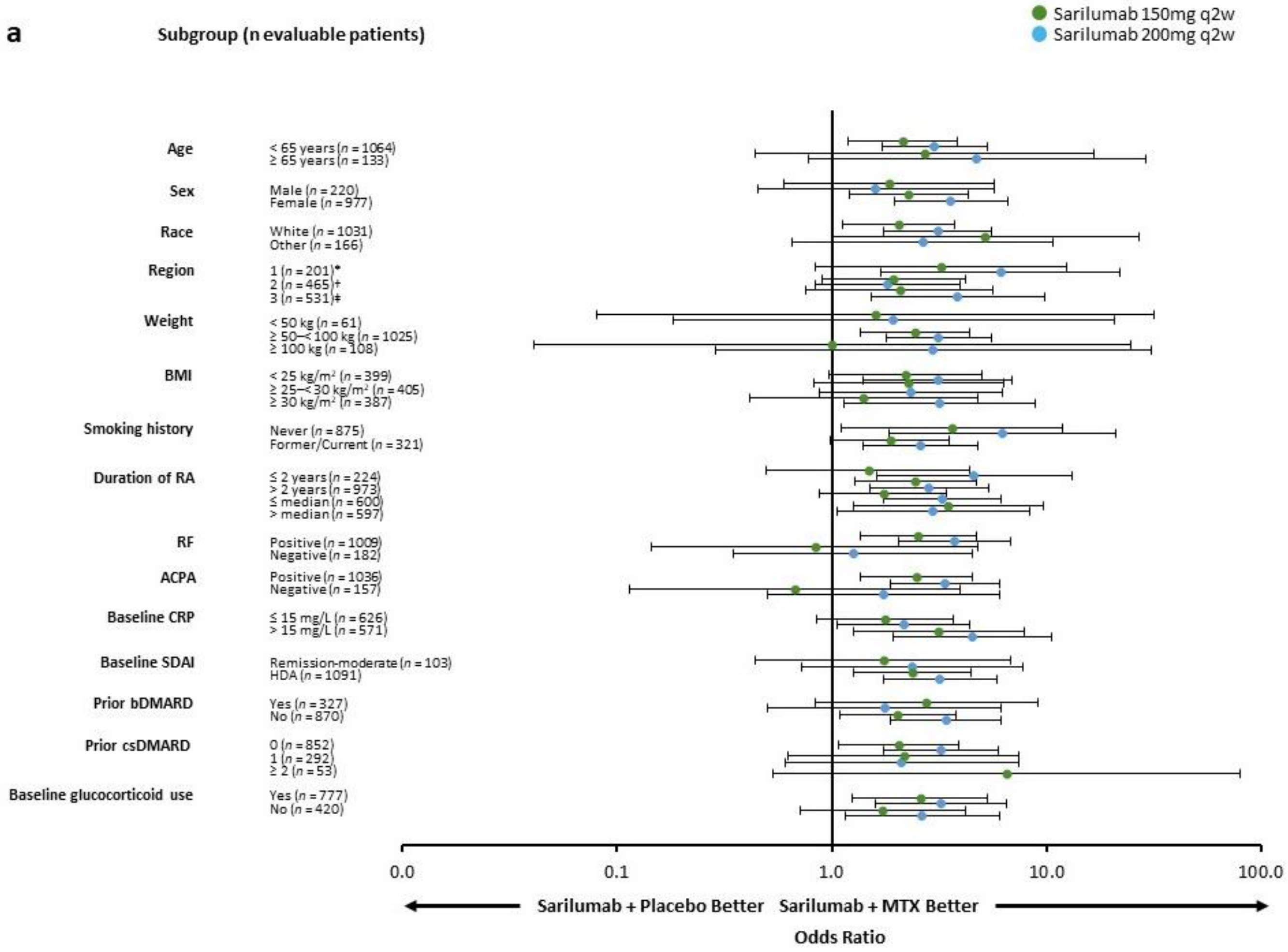

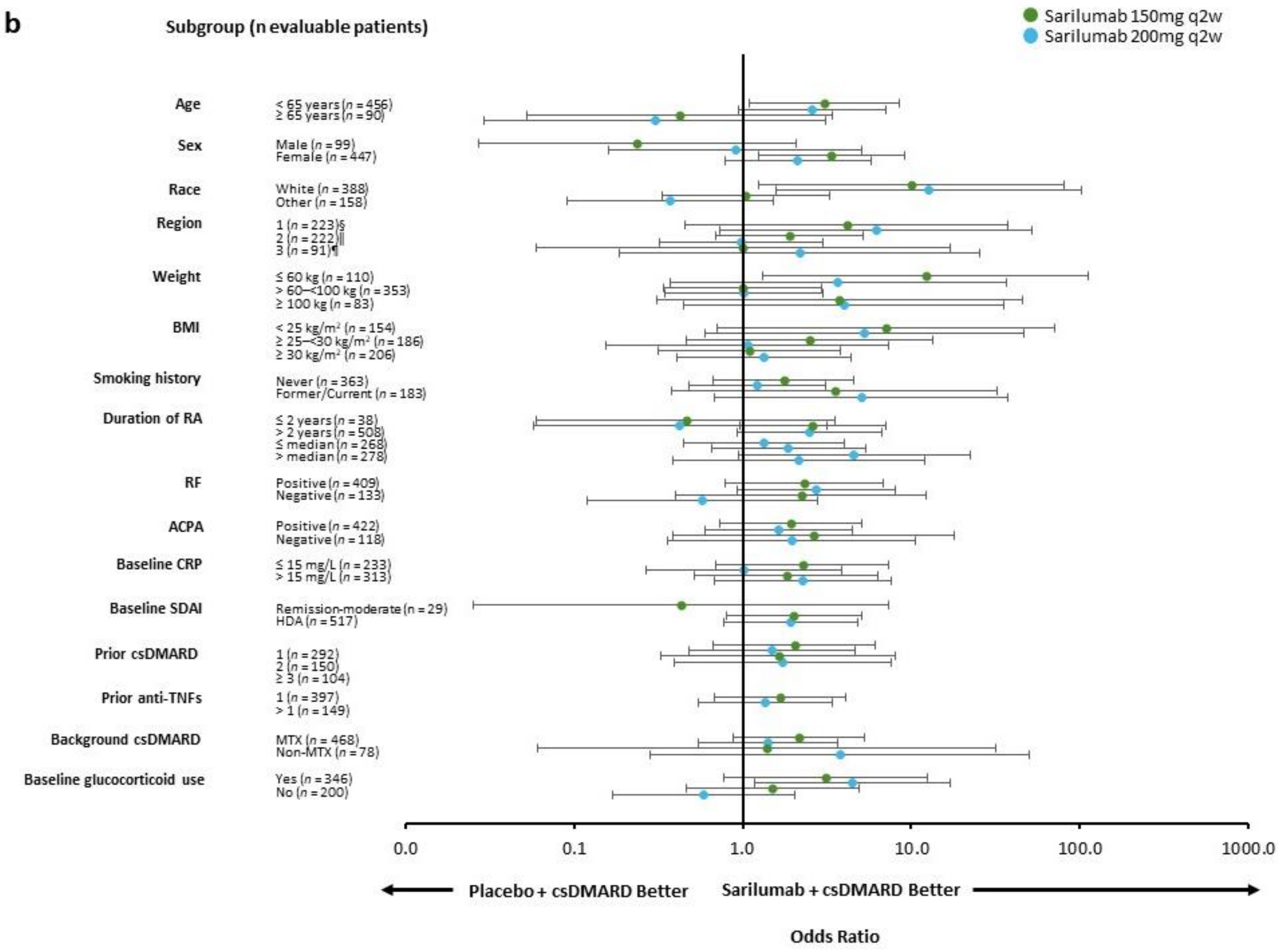
b
Subgroup (n evaluable patients)
Sarilumab 150mg q2w
Sarilumab 200mg q2w
Age
< 65 years (n = 456)
≥ 65 years (n = 90)
Sex
Male (n = 99)
Female (n = 447)
Race
White (n = 388)
Other (n = 158)
Region
1 (n = 223)§
2 (n = 222)||
3 (n = 91)¶
Weight
≤ 60 kg (n = 110)
> 60–<100 kg (n = 353)
≥ 100 kg (n = 83)
BMI
< 25 kg/m² (n = 154)
≥ 25–<30 kg/m² (n = 186)
≥ 30 kg/m² (n = 206)
Smoking history
Never (n = 363)
Former/Current (n = 183)
Duration of RA
≤ 2 years (n = 38)
> 2 years (n = 508)
≤ median (n = 268)
> median (n = 278)
RF
Positive (n = 409)
Negative (n = 133)
ACPA
Positive (n = 422)
Negative (n = 118)
Baseline CRP
≤ 15 mg/L (n = 233)
> 15 mg/L (n = 313)
Baseline SDAI
Remission-moderate (n = 29)
HDA (n = 517)
Prior csDMARD
1 (n = 292)
2 (n = 150)
≥ 3 (n = 104)
Prior anti-TNFs
1 (n = 397)
> 1 (n = 149)
Background csDMARD
MTX (n = 468)
Non-MTX (n = 78)
Baseline glucocorticoid use
Yes (n = 346)
No (n = 200)
0.0
0.1
1.0
10.0
100.0
1000.0
Placebo + csDMARD Better
Sarilumab + csDMARD Better
Odds Ratio

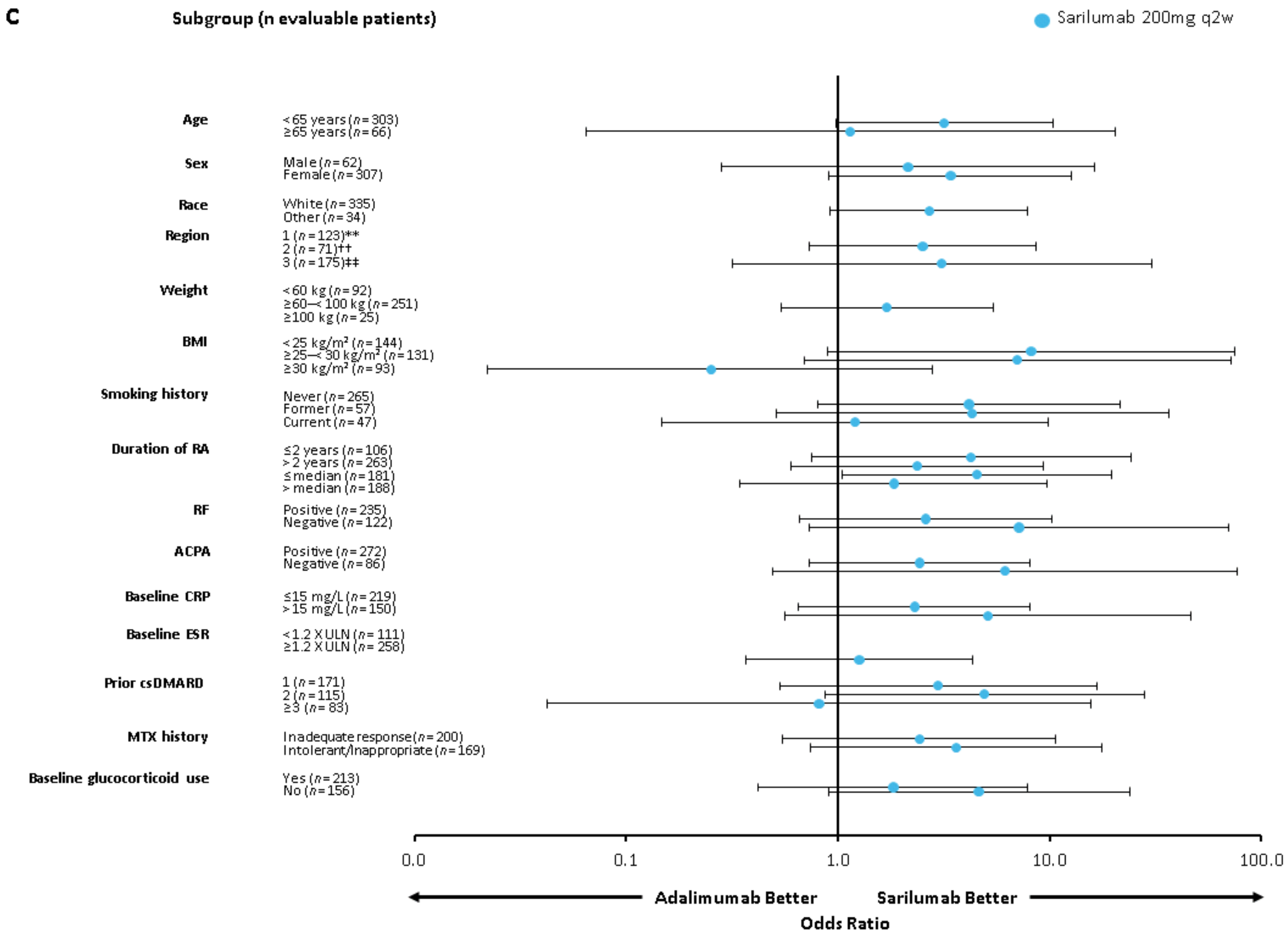

Logistic regression model with terms of (**a**) treatment, prior biologic use, region, subpopulation, treatment-by-subpopulation; (**b**) treatment, prior anti-TNF use, region, subpopulation, treatment-by-subpopulation; (**c**) treatment, region, subpopulation, treatment-by-subpopulation. *ACPA* anti-cyclic citrullinated peptide antibody, *bDMARD* biological and targeted disease-modifying antirheumatic drug, *BMI* body mass index, *CDAI* clinical disease activity index, *CI* confidence interval, *CRP* C-reactive peptide, *csDMARD* conventional synthetic disease-modifying antirheumatic drug, *ESR* erythrocyte sedimentation rate, *HDA* high disease activity, *INT* intolerant, *IR* inadequate response, *MTX* methotrexate, *n* number of evaluable patients regardless of treatment group, *q2w* every 2 weeks, *RA* rheumatoid arthritis, *RF* rheumatoid factor, *SDAI* simplified disease activity index, *TNF* tumour necrosis factor, *ULN* upper limit of normal. *Austria, Australia, Belgium, Canada, Finland, Germany,

Greece, Hungary, New Zealand, Norway, Portugal, Spain, and USA; †Argentina, Brazil, Chile, Colombia, and Mexico; ‡Belarus, Estonia, India, Malaysia, Philippines, Poland, Romania, Russia, South Africa, South Korea, Taiwan, Thailand, and Ukraine; §Australia, Canada, Czech Republic, Germany, Greece, Hungary, Israel, Italy, New Zealand, Portugal, Spain, and USA; ‖Argentina, Brazil, Chile, Colombia, Ecuador, Guatemala, Mexico, and Peru; ¶South Korea, Lithuania, Poland, Russia, Taiwan, Turkey, and Ukraine; **Czech Republic, Germany, Hungary, Israel, Spain, and USA; ††Chile and Peru; ‡‡South Korea, Poland, South Africa, Romania, Russia, and Ukraine.

Figure S5. LSM (95% CI) treatment difference in change from baseline in HAQ-DI by subpopulation. (**a**) sarilumab 150/200 mg q2w + MTX vs. placebo + MTX in MTX-IR patients at week 16; (**b**) sarilumab 150/200 mg q2w + csDMARDs vs. placebo + csDMARDs in TNF-IR/INT patients at week 12; (**c**) sarilumab 200 mg q2w vs. adalimumab 40 mg q2w in MTX-IR/INT patients at week 24

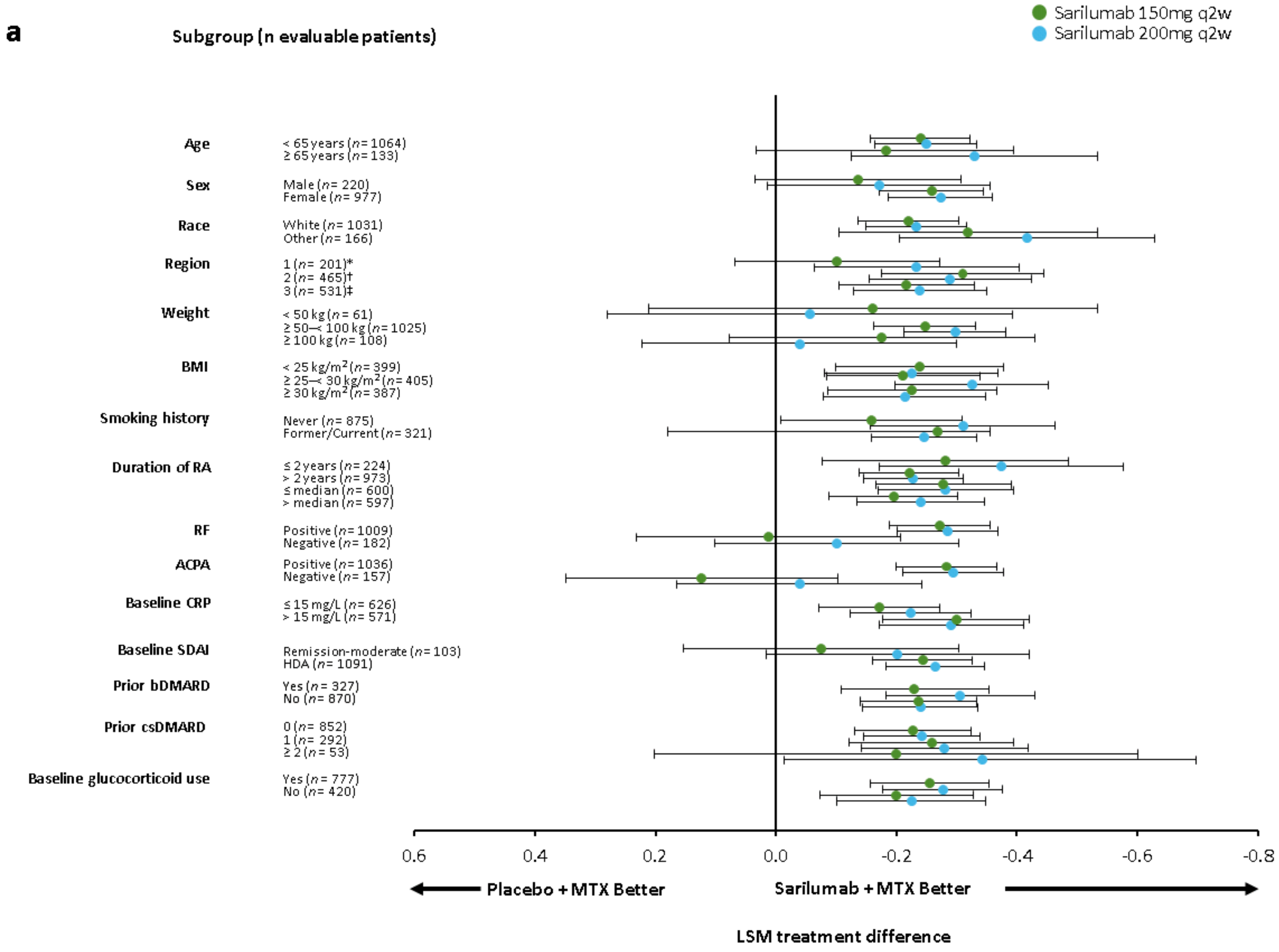

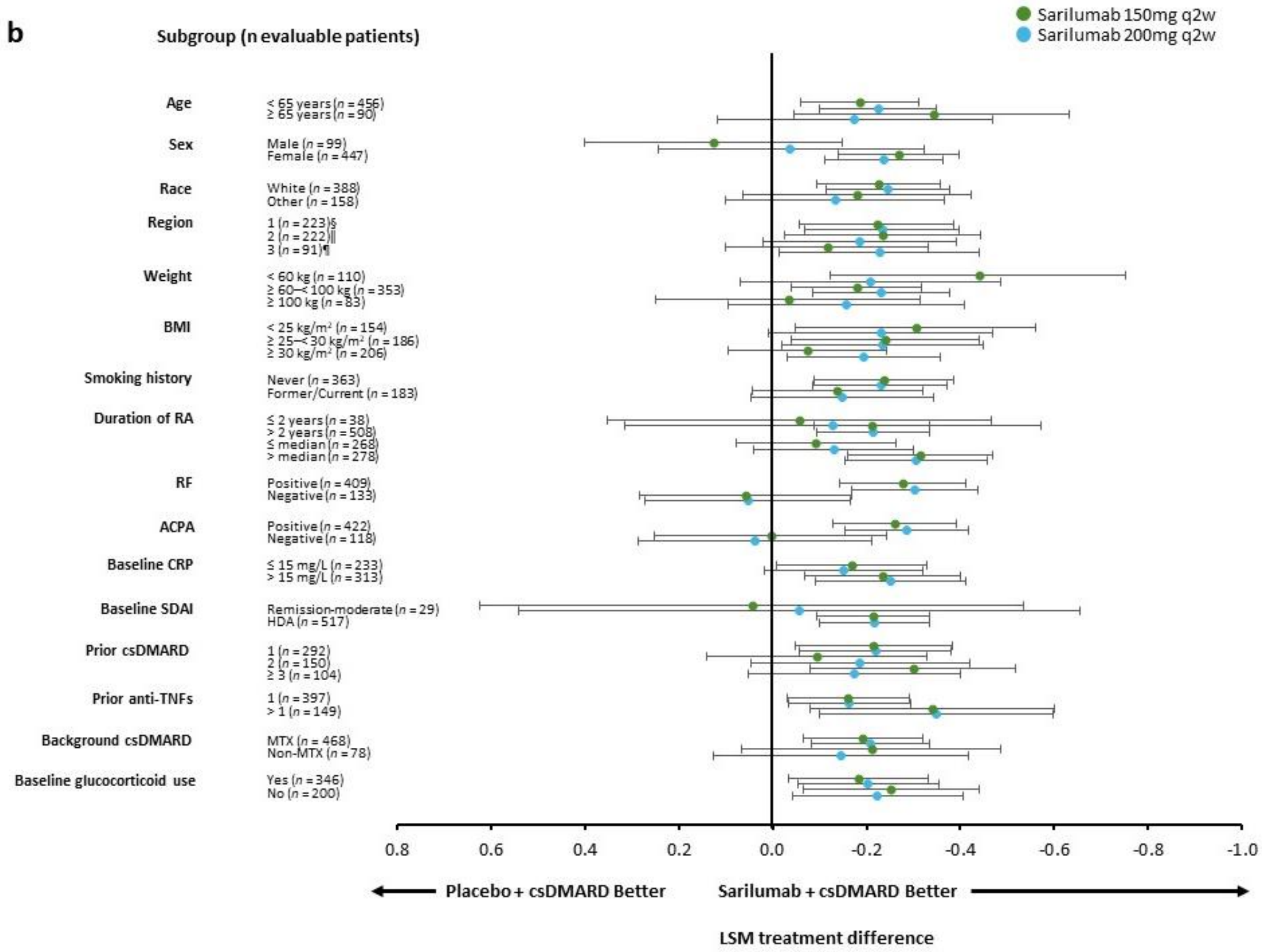
b
Subgroup (n evaluable patients)
Sarilumab 150mg q2w
Sarilumab 200mg q2w
Age
≤ 65 years (n = 456)
≥ 65 years (n = 90)
Sex
Male (n = 99)
Female (n = 447)
Race
White (n = 388)
Other (n = 158)
Region
1 (n = 223)§
2 (n = 222)‖
3 (n = 91)¶
Weight
< 60 kg (n = 110)
≥ 60–< 100 kg (n = 353)
≥ 100 kg (n = 83)
BMI
< 25 kg/m² (n = 154)
≥ 25–< 30 kg/m² (n = 186)
≥ 30 kg/m² (n = 206)
Smoking history
Never (n = 363)
Former/Current (n = 183)
Duration of RA
≤ 2 years (n = 38)
> 2 years (n = 508)
≤ median (n = 268)
> median (n = 278)
RF
Positive (n = 409)
Negative (n = 133)
ACPA
Positive (n = 422)
Negative (n = 118)
Baseline CRP
≤ 15 mg/L (n = 233)
> 15 mg/L (n = 313)
Baseline SDAI
Remission-moderate (n = 29)
HDA (n = 517)
Prior csDMARD
1 (n = 292)
2 (n = 150)
≥ 3 (n = 104)
Prior anti-TNFs
1 (n = 397)
> 1 (n = 149)
Background csDMARD
MTX (n = 468)
Non-MTX (n = 78)
Baseline glucocorticoid use
Yes (n = 346)
No (n = 200)
0.8
0.6
0.4
0.2
0.0
-0.2
-0.4
-0.6
-0.8
-1.0
Placebo + csDMARD Better
Sarilumab + csDMARD Better
LSM treatment difference

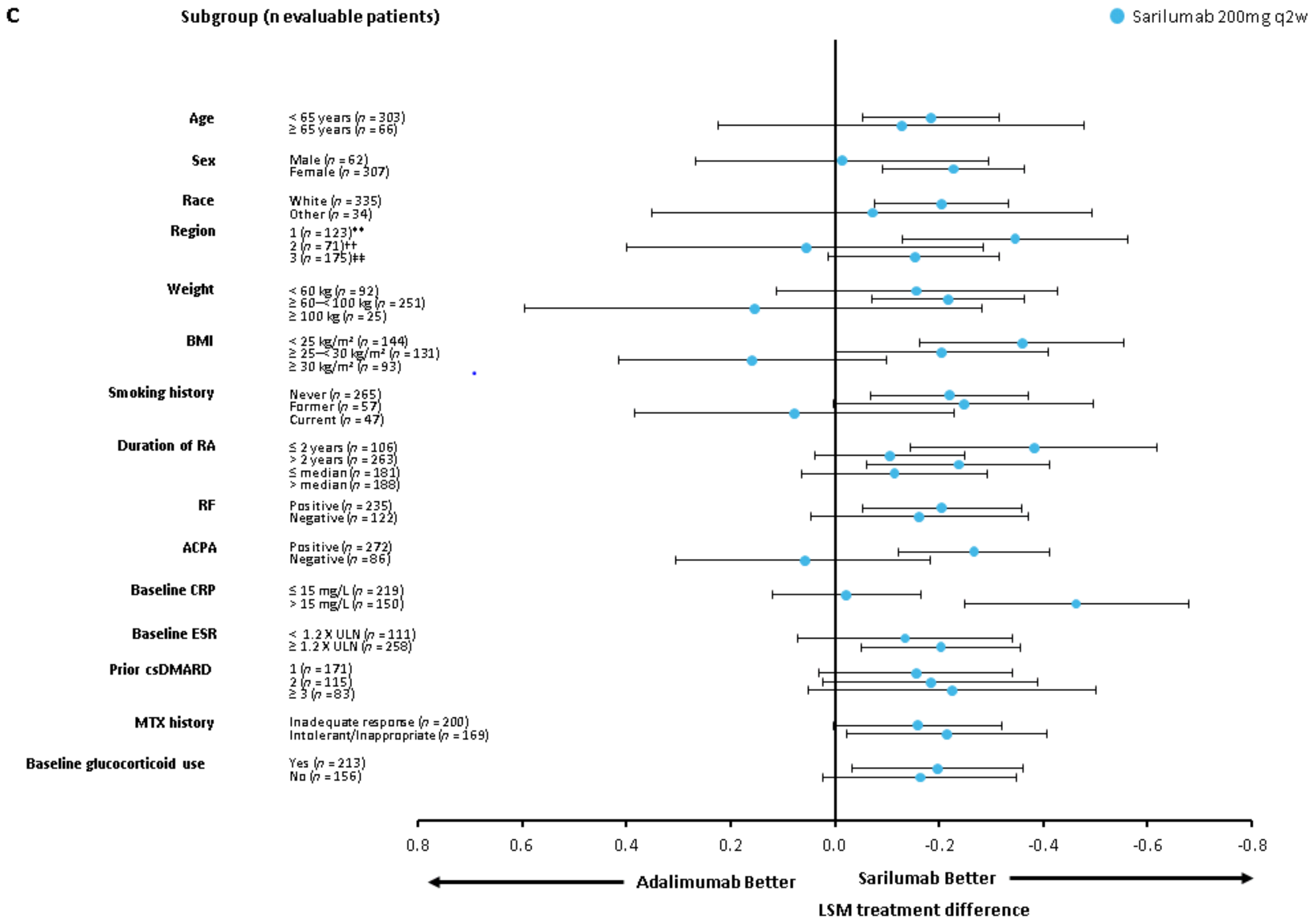

Mixed-effect model for repeated measures with PROC MIXED assuming an unstructured covariance structure: model: (**a**) baseline, treatment, prior biologic use, region, visit, treatment-by-visit interaction (**b**) baseline, treatment, prior anti-TNF use, region, visit, treatment-by-visit interaction. (**c**) baseline, treatment, region, visit, treatment-by-visit interaction. *ACPA* anti-cyclic citrullinated peptide antibody, *bDMARD* biological and targeted disease-modifying antirheumatic drug, *BMI* body mass index, *CI* confidence interval, *CRP* C-reactive protein, *csDMARD* conventional synthetic disease-modifying antirheumatic drug, *ESR* erythrocyte sedimentation rate, *INT* intolerant, *IR* inadequate response, *HAQ-DI* Health Assessment Questionnaire-Disability Index, *HDA* high disease activity, *LSM* least squares mean, *MTX* methotrexate, *n* number of evaluable patients regardless of treatment group, *q2w* every 2 weeks, *RA* rheumatoid arthritis, *RF* rheumatoid factor, *SDAI* simplified disease activity index, *TNF* tumour necrosis factor-α inhibitor, *ULN*

upper limit of normal. *Austria, Australia, Belgium, Canada, Finland, Germany, Greece, Hungary, New Zealand, Norway, Portugal, Spain, and USA; †Argentina, Brazil, Chile, Colombia, and Mexico; [c]Belarus, Estonia, India, Malaysia, Philippines, Poland, Romania, Russia, South Africa, South Korea, Taiwan, Thailand, and Ukraine; ‡Australia, Canada, Czech Republic, Germany, Greece, Hungary, Israel, Italy, New Zealand, Portugal, Spain, and USA; §Argentina, Brazil, Chile, Colombia, Ecuador, Guatemala, Mexico, and Peru; ‖South Korea, Lithuania, Poland, Russia, Taiwan, Turkey, and Ukraine; ¶South Korea, Lithuania, Poland, Russia, Taiwan, Turkey, and Ukraine; **Czech Republic, Germany, Hungary, Israel, Spain, and USA; ††Chile and Peru; ‡‡South Korea, Poland, South Africa, Romania, Russia, and Ukraine.

Figure S6. LSM (95% CI) difference for change from baseline in mTSS for sarilumab 150/200 mg q2w +MTX vs. placebo +MTX at week 52 in MTX-IR patients

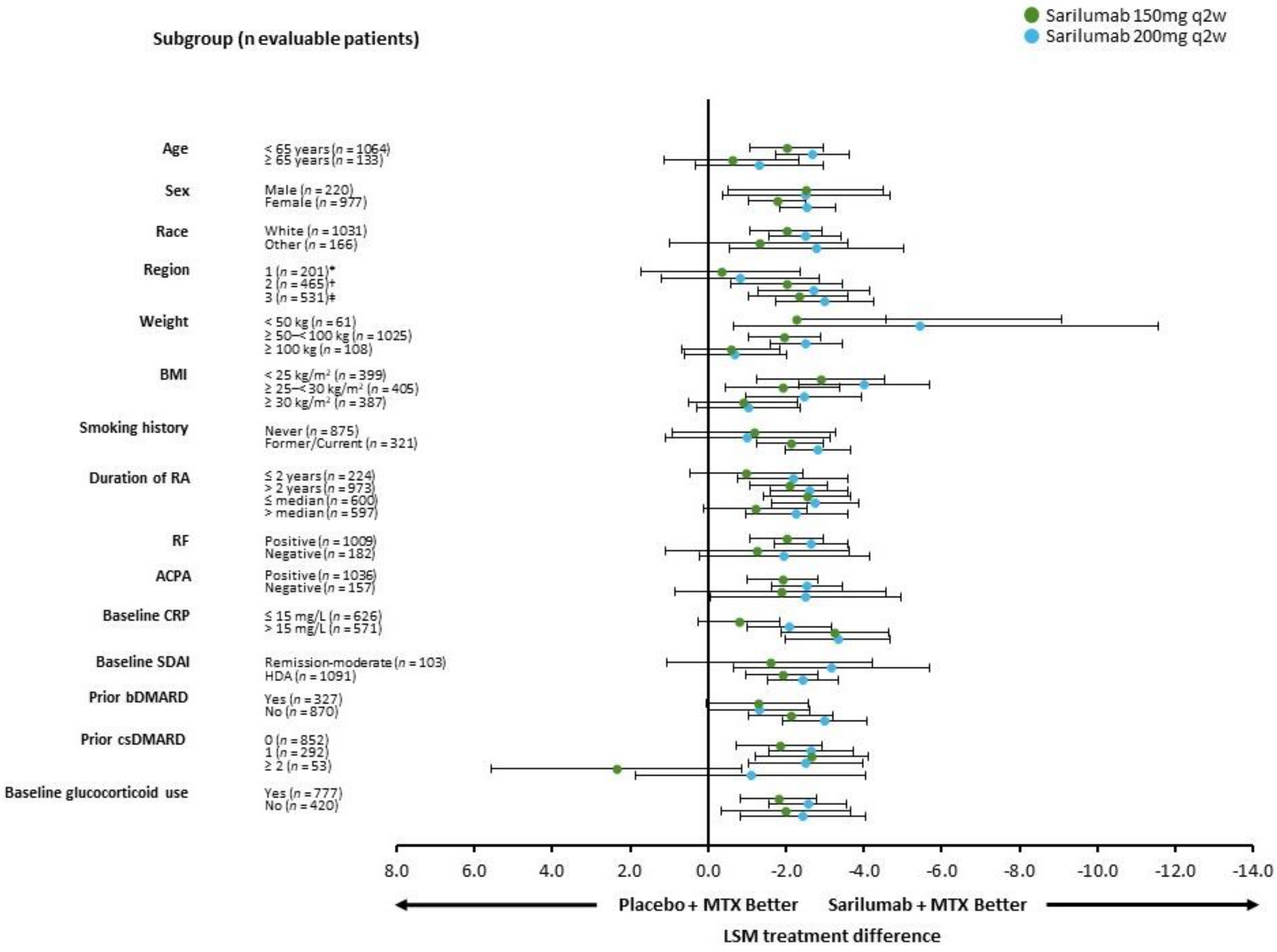

Mixed-effect model for repeated measures with PROC MIXED assuming an unstructured covariance structure: model with baseline, treatment, prior biologic use, region, visit, treatment-by-visit interaction. *ACPA*, anti-cyclic citrullinated peptide antibody, *BMI* body mass index, *bDMARD* biological and targeted disease-modifying antirheumatic drug, *CI* confidence interval, *CRP* C-reactive protein, *csDMARD* conventional synthetic disease-modifying antirheumatic drug, *HDA* high disease activity, *IR* inadequate response, *q2w* every 2 weeks, *LSM* least squares mean, *mTSS* modified total Sharp score, *MTX* methotrexate, *n* number of evaluable patients regardless of treatment group, *RA* rheumatoid arthritis, *RF* rheumatoid factor, *SDAI* simplified disease activity index.
